# Evaluation of femtosecond laser-induced breakdown spectroscopy system as an offline coal analyzer

**DOI:** 10.1038/s41598-021-95317-8

**Published:** 2021-08-05

**Authors:** Sahar Sheta, Zongyu Hou, Yun Wang, Zhe Wang

**Affiliations:** 1 State Key Lab of Power Systems, Department of Energy and Power Engineering, International Joint Lab on Low Carbon Clean Energy Innovation, Tsinghua University, Beijing, 100084 China; 2 Renewable Energy Resources Laboratory, Department of Mechanical and Aerospace Engineering, University of California, Irvine, CA 92697-3975 USA

**Keywords:** Power stations, Optics and photonics, Applied physics, Atomic and molecular physics, Optical physics, Plasma physics, Laser-produced plasmas

## Abstract

Developments in femtosecond laser induced breakdown spectroscopy (fs-LIBS) applications during the last two decades have further centered on innovative métier tie-in to the advantageous properties of femtosecond laser ablation (fs-LA) introduced into LIBS. Yet, for industrially-oriented application like coal analysis, no research has exposed to view the analytical capabilities of fs-LA in enhancing the physical processes of coal ablation and the impact into quantitative correlation of spectra and data modeling. In a huge coal market, fast and accurate analysis of coal property is eminently important for coal pricing, combustion optimization, and pollution reduction. Moreover, there is a thirst need of precision standardization for coal analyzers in use. In this letter, the analytical performance of a one-box femtosecond laser system is evaluated relative to an industrially applied coal analyzer based on five objectives/measures: spectral correlation, relative sensitivity factors, craters topology, plasma parameters, and repeatability. Despite high-threshold operation parameters of the fs system, competitive results are achieved compared to the optimized analytical conditions of the ns-coal analyzer. Studies targeting the *in-field* optimization of fs-LIBS systems for coal analysis can potentially provide insights into fs-plasma hydrodynamics under harsh conditions, instrumental customization, and pave the way for a competitive next-generation of coal analyzers.

## Introduction

Coal energy is used to generate a remarkable chunk of the nation’s electricity and clean coal industry has promoted more attention to an abundant energy source^1,2^. Retrofitting coal and oxy-air in a furnace for optimized combustion requisites coal handling, preparation, and power plants to extensively use quality monitoring systems. Coal quality analysis is performed by several analytical techniques which are classified into two main categories: standards and alternatives. Standard methods are a number of strictly-regulated chemical-based techniques, normally utilize a few grams of coal samples from tons travelling on a conveyor belt, and require several hours. Therefore, standard methods are not able to provide representative results due to the inhomogeneity inherent in coal nor to be qualified for real-time requirements. Alternative methods are physical-based techniques, such as: X-ray fluorescence (XRF), prompt gamma neutron activation analysis (PGNAA), laser-induced breakdown spectroscopy (LIBS), near-infrared spectrometry (NIRS), inductively-coupled plasma atomic emission spectrometry (ICP-AES), atomic absorption spectrometry (AAS), and microwave analysis. Among these, XRF, PGNAA, NIRS, and LIBS are able to provide real-time measurements. LIBS—utilizing nanosecond lasers, has been industrially applied for coal analysis with reasonably simple, robust, and compact analyzers which suit the three installation sets in a power plant: inline, at-line, and offline^3^. Comparatively high sensitivity (unlike XRF), safe operation (unlike PGNAA), and affordable prices (unlike NIRS) forge LIBS as a levelheaded technique for quantitative analysis in coal-fired applications. However, furtherance in measurement accuracy and performance has been one of the important concerns for wide commercialization^4^.

Nanosecond-based LIBS coal analyzers have a long journey with several reported letters on their designs, installation modes (in-line, at-line, and offline), and performance. Inline coal analyzers are directly shooting at coal blocks or pulverized coal over the belt with no sampling system required to feed the analyzer. This is the most convenient way for fast in-situ analysis. However, inline analyzers yield results which suffer from severe measurement errors and high uncertainty. At-line coal analyzers are placed besides the coal flow where samples are made using a sampling system. Offline coal analyzers are usually one-box/unit LIBS systems located near the coal utilization line or at laboratories. The sample is prepared by choosing representative samples from the belt, grinding, mixing, pressing into pellets, analyzing, and finally sending back to the coal stream. Romero et al.^5^ developed an offline coal analyzer to be installed in a power plant and utilized coal samples from 3 mines. Samples were crushed into ~ 250 μm and dried to remove moisture. The unit achieved measurement accuracy for elemental composition within ± 15% (absolute). The unit consists of a ns-ablation source (10 ns pulse width), optical spectrometer, photodiode/amplifier, and processing computer. The sample chamber, machined from aluminum, comprises a sample cart and motorized XY stage, and designed to allow a nonoxygen atmosphere. Data processing was done using artificial neural network models to determine ash fusion temperature with ± 14% °C average precision. Zhang et al.^6^ designed a fully software-controlled LIBS unit with a ns-ablation source (8 ns pulse width), analysis chamber, and a control module. The analysis chamber included a stepping-motor stage and a jet pump to create negative pressure to suck the generated aerosols. A closed-loop feedback laser energy stabilization methodology reduced RSDs of energy variations from ± 5.2% to ± 1.3%. Coal properties; ash content, volatile matter content, and calorific value, were predicted using SVM combined with PCA and the average relative error of prediction reduced from 8.3% to 5.48%, 5.83% to 4.42%, and 5.4% to 3.68%, respectively. On a laboratory level, Redoglio et al.^7,8^ designed a moving system comprised a 500 mm-diameter circular array to house coal samples and rotate at fixed speed. The optical system with a large depth-of-field mirror was utilized to overcome the changing in coal rocks’ height. This combination of sample rotating tray and optical system allowed scanning coals at desired sampling frequency to cope with the in-line requirements of analysis. A close design was employed by Gaft et al.^9,10^ at LDS LIBS unit (in-line mode of operation) to overcome the variation of coal height using an ultrasonic sensor which moves the entire optical system to maintain the focal length of the focusing lens. The different designs in literature have proved useful in providing technical specifications for LIBS customization in coal industry. Along with the ameliorating of data modeling, a wide research direction is opened for next generations of LIBS coal analyzers with ultrafast lasers as ablation sources.

In fact, progress in analytical techniques based on laser ablation (LA) continually places demands on developing informative analytical methods with competing performances for different applications. Ultrashort pulse lasers (pulse width < 1 ps) have addressed fundamental changes in the ablation process due to different mechanisms of power dissipation. LIBS, as an analytical technique widely used for solid sampling, benefits by femtosecond lasers as ablation sources and improves its figures of merit with: enhanced repeatability^11^, reduced matrix effects^12^, better depth control and quality crater^13^, and reduction in damage^14,15^. At present—to the authors’ knowledge, only two references to date make use of fs-LIBS systems to coal analysis. Hemalaxmi et al.^16^ detected C, Al, Fe, and Ca in coal and ash samples using fs-LIBS system. The carbon content was found to be correlated to the C_2_ and CN molecular bands. Jian et al.^17^ combined fs-LIBS with PLS model to quantitatively determine the calorific heat value in coal samples. Authors claimed that when the fs laser is used as the ablating source and a ns laser is used for heating plasma, the dual-pulse system would improve the quantification results. Despite useful results to this end, no introductory study has evaluated ultrafast lasers as innovative ablation sources for coal analysis in comparison to industrially-applied coal analyzers. This approach paves the way to methodical optimization studies from a technical point of view; necessary to provide solutions for LIBS analytical tasks in industry.

In this letter, a fs-LIBS system is evaluated in comparison to industrially applied coal analyzer. The concept described in this paper evolves in steps to cover five objectives for a performance measure that starts with emphasizing the differences between fs and ns-LAs analytical capabilities to represent coal structure by analyzing lines with highest correlation coefficients to coal properties. Molecular fragmentation and ionization are discussed to understand the high representativeness and selectiveness of lines within molecular bands correlated to coal properties in case of fs-LA. Ionic and atomic lines abundancies in fs-plasma are tested by calculating relative sensitivity factors. Coulombic regime of plasma hydrodynamics in fs-LA incurs fast dissipation of pulse energy density where electrostatic ejection and formation of atomic, ionic, and molecular fragments are dominant. Craters morphologies are examined to explain the enhanced laser-energy coupling in case of fs-LA. Plasma temperatures and electron number densities are calculated and the cooler ablation of fs plasmas is emphasized. Later, spectral repeatability is accessed by average measurement-to-measurement relative standard deviations (RSDs) of raw spectral lines. At the end of this letter, a discussion about the applicability and problems of fs-LIBS systems for industrial applications is conducted, along with the evaluation results and summary.

## Results and discussion

### Correlation coefficients

**Figure 1:**
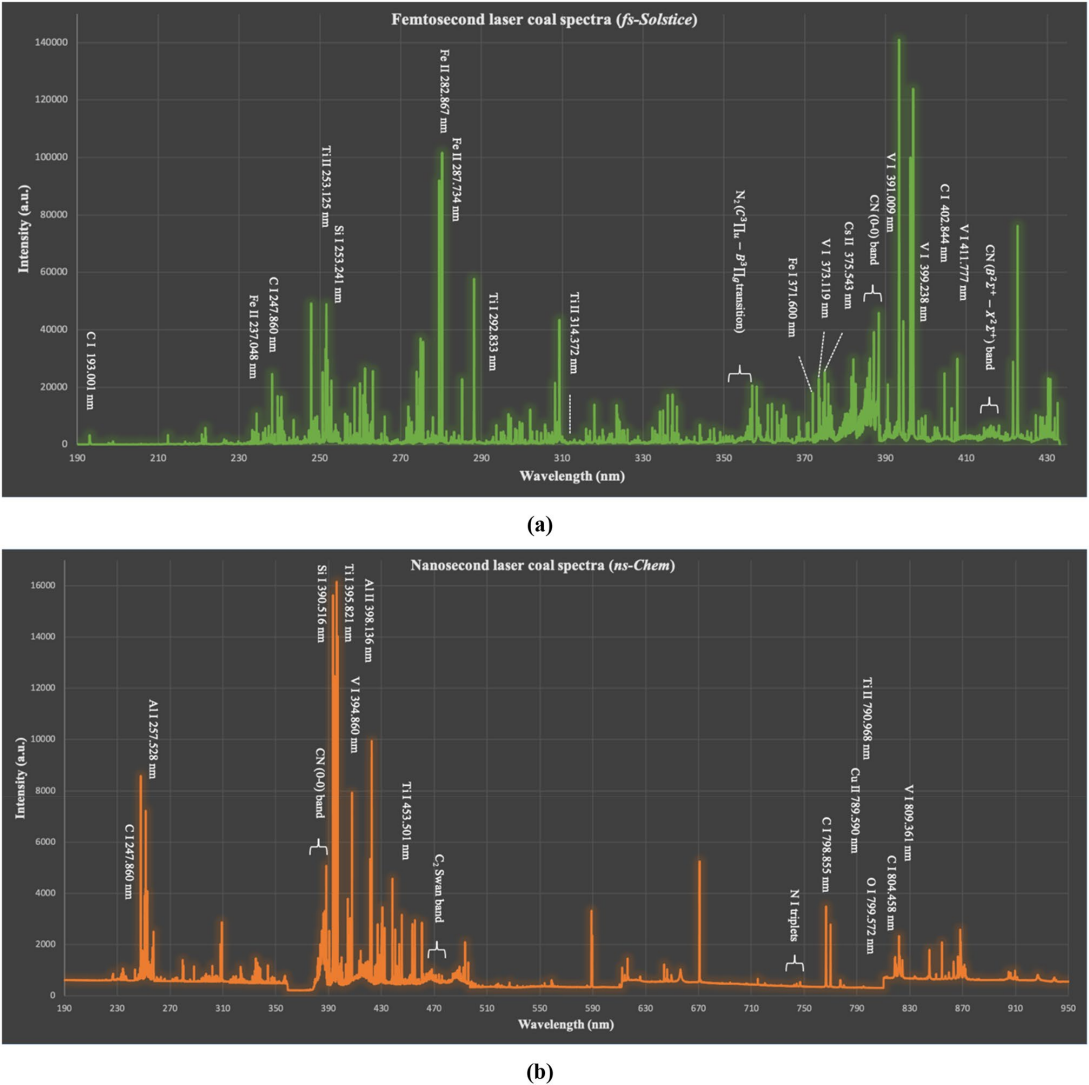
Coal spectra obtained by *fs-Solstice* system in (**a**) and *ns-Chem* system in (**b**). A plentiful population of atomic, ionic, and molecular lines in both spectra indicates the enrichment of plasma species in the fs and ns ablation conditions.

Direct correlation between quantitative information of coal spectra and coal analysis is one of the tentative and tricky applications of LIBS^18^. The situation is complicated due to: matrix complexity and property interdiscursivity of coal. The heterogeneous composition of coal, with a mixture of organic and inorganic molecules which are considerably varying in size and structure^19^, implements fluctuated plasma kinetics and spectral interdependency. The coal properties interdiscursivity is simply noticed by calorific heat value, volatile, and ash contents definitions. Caloric heat value is the net heat released from coal combustion with oxygen. It integrates the combustion heat of C, H, O, S, ash, and volatile matter contents. Volatile matter includes long-chains hydrocarbons, aromatic hydrocarbons, and sulfur. Ash includes the noncombustible residue of mineral oxides and sulfates. Therefore, major and mineral lines carry spectral information related to different coal properties. In this work, a total of 998 and 900 lines are observed by the fs and ns LIBS systems, respectively (denoted as *fs-Solstice* and *ns-Chem—*see materials and systems section for more details). The congestion of the coal spectra due to abundance of mineral elements makes emission lines identification a cumbersome task and misidentification of lines is easily probable. Therefore, spectra from *fs-Solstice* and *ns-Chem* systems were carefully identified using NIST database^20^ and confirmed by literature. A plentiful population of atomic, ionic, and molecular lines are observed in both spectra indicating the enrichment of plasma species in fs and ns ablation conditions as shown in Fig. 1.

This expected congestion of coal spectra means that the normal situation of extracting coal properties from specified spectral lines might limit the analysis accuracy due to stepwise correlations^21^. Consequently, several multivariate and hybrid models^22–24^ have been used to extract correlated spectral information to concentrations. These models, still and all, provide robust algorithms to enhance quantitative measurements for a number of applications, but have degraded accuracy for materials with heterogenous/matrix composition like coals^3^. With increasing chemical complexity, algorithms functionalize with solely statistical correlations or curve overfitting eventually ruin the measurement trueness due to lack of physical picture of the fingerprint emissions from parent molecules in the LA process. To this extent, it is significant to emphasize the analytical capabilities of fs-LA to represent coal structure by analyzing lines with highest correlation coefficients to coal properties relative to those obtained by the optimized *ns-Chem* system. Pearson correlation (***r***) is commonly used in linear regression to measure a relationship strength between two variables. It is given by the formula^25^:

1 $${\varvec{r}}= \frac{\sum \left(x-\overline{x }\right)\left(y-\overline{y }\right)}{\sqrt{\sum {\left(x-\overline{x }\right)}^{2}{\left(y-\overline{y }\right)}^{2}}}$$

where; ***r*** is the correlation coefficient, *x* is the standard value and *y* is the line intensity. The bar sign shows the average value of all measurements.

**Figure 2:**
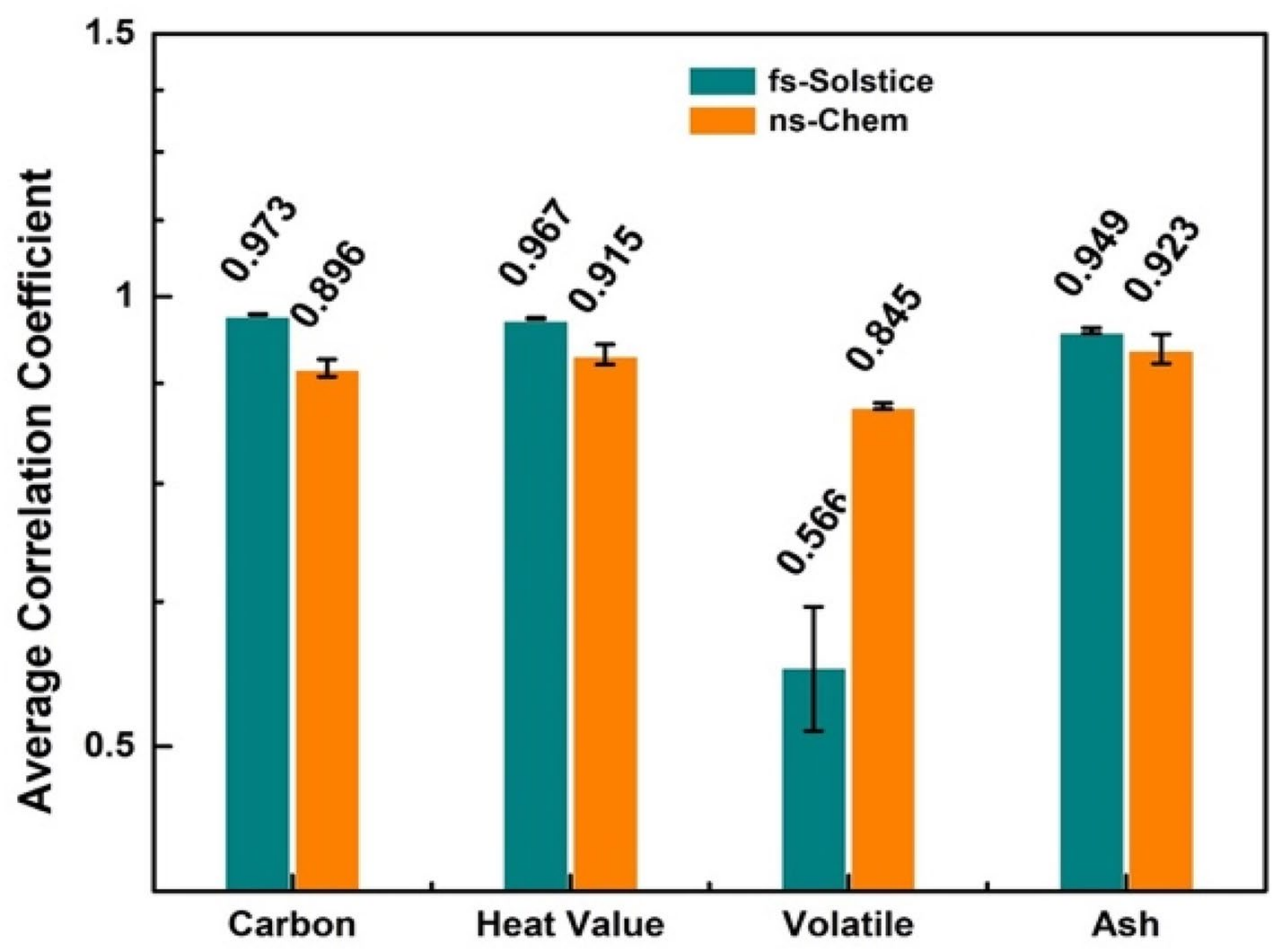
Average of Pearson’s correlation coefficients of 10 most correlated lines to carbon content, heat value, volatile, and ash contents obtained by *fs-Solstice* and *ns-Chem* systems.

Pearson correlation analysis has been used for the identification, classification, and determination of physical/chemical properties of different materials relative to reference spectra. However, to ensure correct and fair correlation magnitude of ***r***, a few factors have to be considered: (1) Essential spectra related to laser-matter interaction and probably to a surrounding gas have to be included while non-essential spectral information (random noise) have to be excluded; (2) Sufficient number of observations (well-resolved and non-saturated lines) should be available in correlated spectra to confirm correct representativeness of ***r***; (3) Multiple sampling is essential to avoid misleading ***r*** values due to strongly nonlinear character of a combination of factors such as the laser-matter interaction, surface roughness, sample heterogeneity, etc^26^. Considering those factors, Gornushkin et al.^27^ used Pearson correlation for the identification of glass samples of forensic interest. The spectral data was utilized after two pre-processing steps of: rejection of spectral outliners (filtering) and removal of spectral fragments which contain no correlated information (masking). The proposed procedure showed 100% identification rate. Lentjes et al.^28^ found that averaging the spectra enhanced the correlation accuracy from 79% for single shot to 99.9% for average spectra. Therefore, during our work, multi-pulse averaging, background subtraction, and normalization by the whole spectral area were applied as pre-processing methods to compensate for variations in laser energies and to ensure fair ***r*** values for each system. Visual inspection of spectral lines was done to ensure that the lines which showed highest correlation are non-saturated lines, well-resolved, and have stable intensity over the 40 samples (validity for analytical use). Figure 2 shows average correlation coefficients of 10 most correlated lines for carbon content, heat value, volatile, and ash contents using *fs-Solstice* and *ns-Chem* systems. The error bars are standard deviations of 40 measurements corresponding to 40 samples in use. The correlation coefficients of carbon, heat value and ash are $\ge$ 0.949 for *fs-Solstice* and $\le$ 0.923 for *ns-Chem* showing higher representativeness of the fs spectra to coal property. For volatile matter content, the fs-spectra show correlation of 0.566 which is lower than 0.845 for the ns-spectra. The identification of the correlated lines for each coal property may uncover reasons behind low ***r*** values in case of volatile matter content and reveal differences in ablation mechanisms for *fs-Solstice* relative to *ns-Chem.*

Table 1 illustrates the identification and evaluation of the 10 most correlated lines for carbon content. The correlation coefficients vary between 0.975 and 0.971 for *fs-Solstice*, while for *ns-Chem*, correlation coefficients show larger variations between 0.907 and 0.879 for the 10 most correlated lines. For *fs-Solstice* spectra, seven lines are identified^29^ within the CN (${\mathrm{\rm B}}^{2}{\Sigma }^{+}-{\mathrm{\rm X}}^{2}{\Sigma }^{+}$) violet system ($\Delta \nu =-1$), while three lines are Fe I and V I mineral lines. For *ns-Chem* spectra, two C I lines at 798.8 and 804.4 nm show high correlation with carbon content. Other organic lines are an O I line at 799.5 and N I triplet at 742.4, 744.3, and 746.9 nm. Residuals are four mineral lines of V I, Cu II, Ti II, and Cs II. Same lines with different correlations order show ***r*** values ranged between 0.924 and 0.894 to caloric heat content as shown in Table 2. While for the *fs-Solstice* spectra, four lines are identified within the CN molecular band where two of them correlate to heat value and not to carbon content showing higher selectiveness of the fs-LA relative to its ns-counterpart. Another four lines are unforeseen emissions from the second positive band system^30,31^ (${\mathrm{C}}^{3}{\prod }_{u}-{\mathrm{B}}^{3}{\prod }_{g}$ transition) of N_2_. It means that 7/10 and 8/10 of the most correlated lines for carbon content and heat value respectively are molecular lines for *fs-Solstice* spectra.

**Table 1:** Evaluation of *fs-Solstice* spectra correlation to carbon content in coal samples.

Carbon content						Evaluation
*fs-Solstice*			*ns-Chem*			
λ/nm	*r*	Line	λ/nm	*r*	Line	
417.645	0.975	CN	798.855	0.907	C I	Higher representativeness of *fs-Solstice* spectra (larger ***r*** values)
417.953	0.974	CN	804.458	0.906	C I	
371.600	0.974	Fe I	809.361	0.904	V I	
417.526	0.973	CN	799.572	0.904	O I	
418.240	0.973	CN	789.590	0.903	Cu II	
417.189	0.973	CN	790.968	0.903	Ti II	
415.808	0.972	CN	793.434	0.902	Cs II	
411.777	0.972	V I	744.322	0.882	N I	
415.709	0.972	CN	742.480	0.881	N I	
373.119	0.971	V I	746.971	0.879	N I

**Table 2:** Evaluation of *fs-Solstice* spectra correlation to heat value in coal samples.

Heat value						Evaluation
*fs-Solstice*			*ns-Chem*			
λ/nm	*r*	Line	λ/nm	*r*	Line	
415.808	0.970	CN	798.855	0.924	C I	Higher selectiveness of *fs-Solstice* spectra (different molecular fragments correlated)
375.543	0.968	Cs II	789.590	0.924	Cu II	
415.236	0.968	CN	809.361	0.923	V I	
356.533	0.968	N_2_^+^	804.458	0.923	C I	
355.280	0.968	N_2_^+^	790.968	0.923	Ti II	
354.413	0.968	N_2_^+^	793.434	0.921	Cs II	
373.119	0.968	V I	799.572	0.921	O I	
418.240	0.968	CN	744.322	0.896	N I	
356.204	0.968	N_2_^+^	746.971	0.894	N I	
415.674	0.967	CN	742.480	0.894	N I	

**Figure 3:**
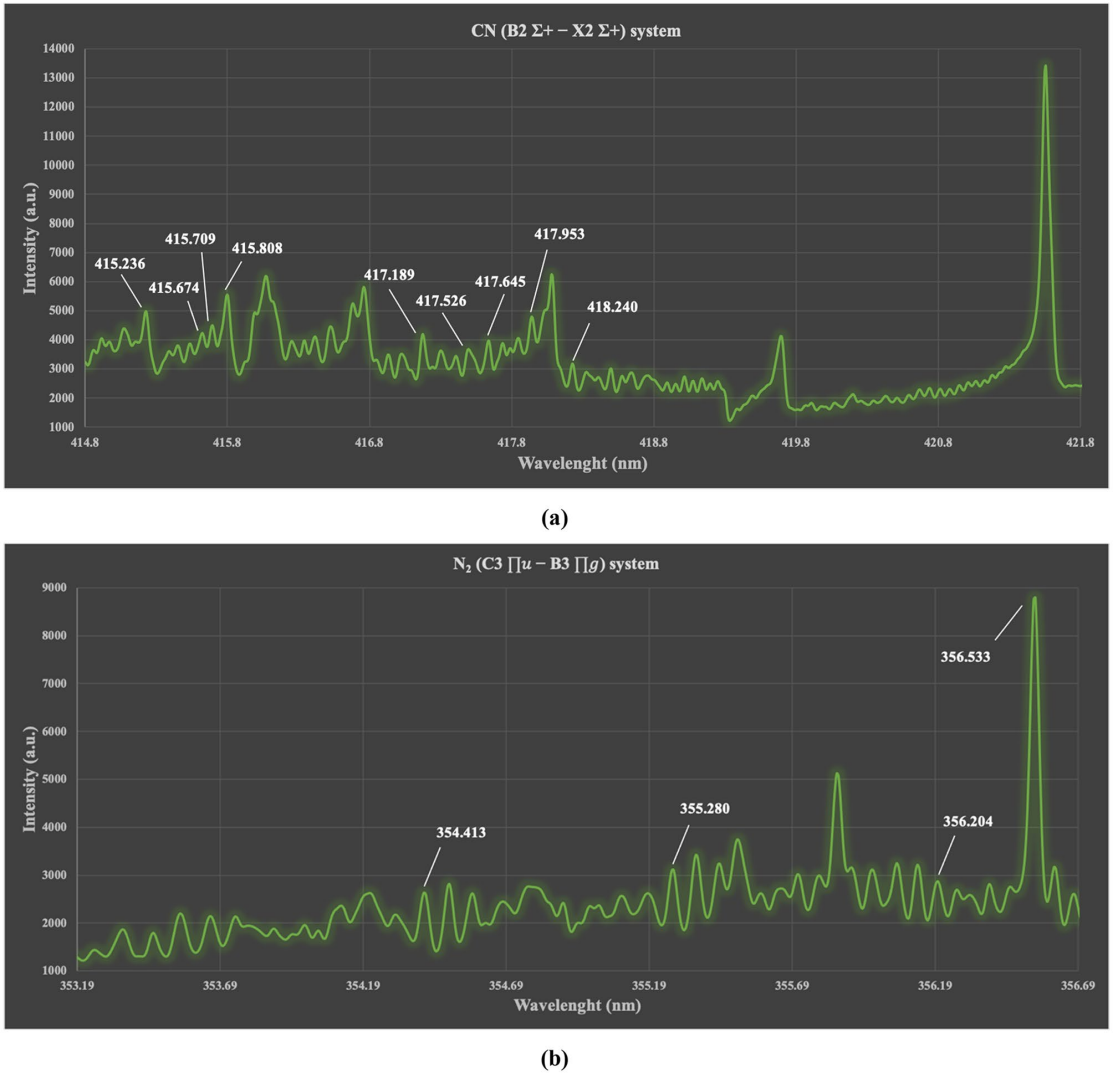
The CN (${\mathrm{\rm B}}^{2}{\Sigma }^{+}-{\mathrm{\rm X}}^{2}{\Sigma }^{+}$) and the N_2_ (${\mathrm{C}}^{3}{\prod }_{u}-{\mathrm{B}}^{3}{\prod }_{g}$) molecular systems detected into *fs-Solstice* spectra of coal and lines within which show high correlations to carbon content and/or heat value.

During the last few years, molecular fragmentation and ionization have been evolved significantly in molecular sensing studies as fundamental interaction processes in fs-LA^32,33^ of organic materials. Due to the short-time interaction of super intense laser fields, the geometrical structure of molecules becomes deformed within ~ 100 fs and tunneling ionization occurs. Charged molecules are formed triggered by strong Coulombic repulsive force and abrupt chemical bond fission occurs where atomic and ionic species with large kinetic energies are ejected. In collisional environment like air, resultant ions/fragments undergo random scattering/collision reactions in a relatively slower time scale leading to optical emissions. In an ideal reaction process; i.e. collision-less, fs-LA molecular ejecta is typically fingerprinting the parent molecules. The formation pathways of CN molecular bands in fs-based interaction zone have been discussed under three major formation mechanisms: direct fragmentation from species containing CN bonds in its structure at early stages of plasma evolution near surface (native CN), recombination of atoms from compounds in plasma to produce CN molecules (collisions-CN), and reactive atomic recombination of C from plasma with N from ambient or air (ambient-CN). For the 2^nd^ and 3^rd^ pathways, CN molecules’ detection is sensitive to spatiotemporal conditions of the plasma. The persistence of CN molecular bands in fs-LAs is well reported. Baudelet et al.^34^ investigated native CN molecular bands of bacteria where the band-head intensity reached maximum at 450 ns delay time. Zhao et al.^35^ found that the native CN band-head persisted before 700–800 ns delay time (the delay time chosen in our experiment is 400 ns). As for the nitrogen band, the N_2_^+^ fragments could come from^36^: collision of resultant fragments (NH radicals) within the dense plasma or with ambient particles or with energetic electrons. Since N–N bond structure might not exist in coals, N_2_^+^ fragments are probably formed due to ionization of air by energetic electrons at the interaction zone on coal surface—which is the claim behind enhanced residual thermal energy deposition explained later in this paper. Yet, the dynamical reactions of hydrocarbons in air are still under investigation^37^. Figure 3 shows the CN (${\mathrm{\rm B}}^{2}{\Sigma }^{+}-{\mathrm{\rm X}}^{2}{\Sigma }^{+}$) and the N_2_ (${\mathrm{C}}^{3}{\prod }_{u}-{\mathrm{B}}^{3}{\prod }_{g}$) molecular systems detected into *fs-Solstice* spectra of coal and show high correlations to carbon content and heat value.

As for volatile matter content, Table 3 depicts the lines of *fs-Solstice* and *ns-Chem* spectra which show highest correlations to volatile matter content. For *ns-Chem* spectra, lines are detected within the C_2_ swan system (${d}^{3}{\prod }_{g}-{a}^{3}{\prod }_{u}$) and ***r*** values are in the range 0.853–0.841 (weak correlation values). As a consensus reality, volatile matter content determination is a hectic task due to limitations related to the accuracy of the eventual detection of elements integrating spectral information to the property content. In a sense, volatile matter is standardly measured as the weight percent of gas (emissions) that is released during heating a dried coal sample to 950 °C in an oxygen-free environment by thermogravimetric analysis (TGA). The integrated intensities of C, H, O, N, and S lines are hypothetically representing correlated data set to volatile content. However, O and N can be originating from the surrounding air and reaction of O with minerals in coal to form ash have to be considered during data handling. Moreover, sulfur is volatilized as sulfur dioxide with hardly detectable lines in many experimental protocols. Yao et al.^38^ discussed coals with different volatile contents and found that changes in volatile content ushered different emission line intensities and spatial distribution of major and mineral elements in the evolved ns-plasma. Several experimental and data modeling methods have been discussed in literature to enhance volatile matter content measurement by LIBS. We refer to the collection of works summarized in the analytical results section of the tutorial review by Sheta et al.^3^. For *fs-Solstice* spectra, weaker correlation is detected (***r*** values are in the range 0.704–0.518) than those of the reference spectral set*.* Despite 6/10 of the most correlated lines are CN and C I lines, it may just be a statistical artifact instead of based on real physical background. Worth to mention that the molecular band correlated to volatile content by the *ns-Chem* spectra is out of the spectrometer range for the *fs-Solstice* system. The importance of establishing an accurate correlation between spectral data set and volatile matter content urges attention to further studies based on understandable interpretation of LA-physics. For ash content, Table 4 collects the identified lines correlated to the property. Both spectra revealed well-resolved, non-saturated, and mineral lines of Ti, Si, and Fe with larger ***r*** values detected for *fs-Solstice* lines. Unexpectedly, mineral lines most correlated to ash content are mainly ionic for *fs-Solstice* spectra and atomic for *ns-Chem* which is contrast to the claim of fs-plasmas atomic nature^39,40^. The evaluation of atomic and ionic lines abundance in *fs-Solstice* spectra would be investigated in the next performance measure of the conducted evaluation study.

**Table 3:** Evaluation of *fs-Solstice* spectra correlation to volatile content in coal samples.

Volatile content						Evaluation
*fs-Solstice*			*ns-Chem*			
λ/nm	*r*	Line	λ / nm	*r*	Line	
314.372	0.704	Ac I	473.599	0.853	C_2_	Weak correlation of both *fs-Solstice* and *ns-Chem* (reference data set) spectra
387.646	0.585	CN	471.403	0.848	C_2_	
387.692	0.575	CN	468.383	0.846	C_2_	
391.009	0.573	V I	470.332	0.845	C_2_	
387.588	0.567	CN	470.131	0.844	C_2_	
399.238	0.549	V I	469.662	0.844	C_2_	
402.844	0.535	C I	470.667	0.842	C_2_	
373.964	0.526	Pb I	470.466	0.842	C_2_	
387.744	0.524	CN	471.002	0.841	C_2_	
193.001	0.518	C I	469.997	0.841	C_2_

**Table 4:** Evaluation of *fs-Solstice* spectra correlation to ash content in coal samples.

Ash content						Evaluation
*fs-Solstice*			*ns-Chem*			
λ/nm	*r*	Line	λ/nm	*r*	Line	
253.125	0.955	Ti II	395.821	0.949	Ti I	High correlation of both *fs-Solstice* and *ns-Chem* spectra
292.833	0.954	Ti I	395.661	0.947	Fe I	
237.048	0.952	Fe II	394.860	0.943	V I	
282.867	0.950	Fe II	399.886	0.929	Ti I	
287.734	0.950	Fe I	398.136	0.927	Al II	
237.517	0.948	Fe II	398.933	0.922	Ti I	
226.005	0.947	Fe II	453.501	0.916	Ti I	
236.923	0.945	Fe II	453.289	0.910	Ti I	
309.712	0.943	Ti II	390.516	0.896	Si I	
253.241	0.943	Si I	257.528	0.885	Al I	

### Relative sensitivity factor

With a few exceptions, minerals are the prim hosts of vast majority of elements in coals^41^. On the basis of their concentration, mineral elements are considered minor and trace elements. However, they are easily atomized and ionized in coal matrix like Fe, Si, Mg, Al, Ti, and Ca and attribute to higher emissions from matrixes and early stages of plasma formation^42^. Mineral elements affect, straightforwardly, the detection accuracy of volatile and ash contents, and the plethora of their lines in coal spectra plays a key role in full spectral information applied in several multivariate algorithms for enhanced quantitation. Relative sensitivity factor (RSF) is used to measure the spectral detection discernment of an element by intensity/concentration ratios normalized to an internal standard element. In other words, RSFs correct for the differences in the response to several elements by a specific technique, as well as for changes in the response to a species due to changes in matrix. A wide variety of analytical techniques, viz. glow discharge mass spectroscopy^43^ and secondary ion mass spectrometry^44^, rely on the use of RSFs for obtaining semi-quantitative results for multicomponent samples. In this work, RSFs have been determined for the analysis of 6 mineral elements from multiple analyses of the 40 standard coal samples representing different matrices for *fs-Solstice* system relative to *ns-Chem* system. As each element has a fixed concentration in a specific sample, the sensitivity response of the fs system is measured with RSF, which is determined as follows^45^:

2 $${\text{RSF }} = \, \left( {{\text{I}}_{{\text{x}}} /{\text{I}}_{{\text{M}}} } \right)_{{{\text{fs}}}} / \, \left( {{\text{I}}_{{\text{x}}} /{\text{I}}_{{\text{M}}} } \right)_{{{\text{ns}}}}$$

where; x is the element of interest and M is an internal standard element. Since carbon is presented in all coal samples, it was chosen as the internal standard element for RSF calculations.

**Figure 4:**
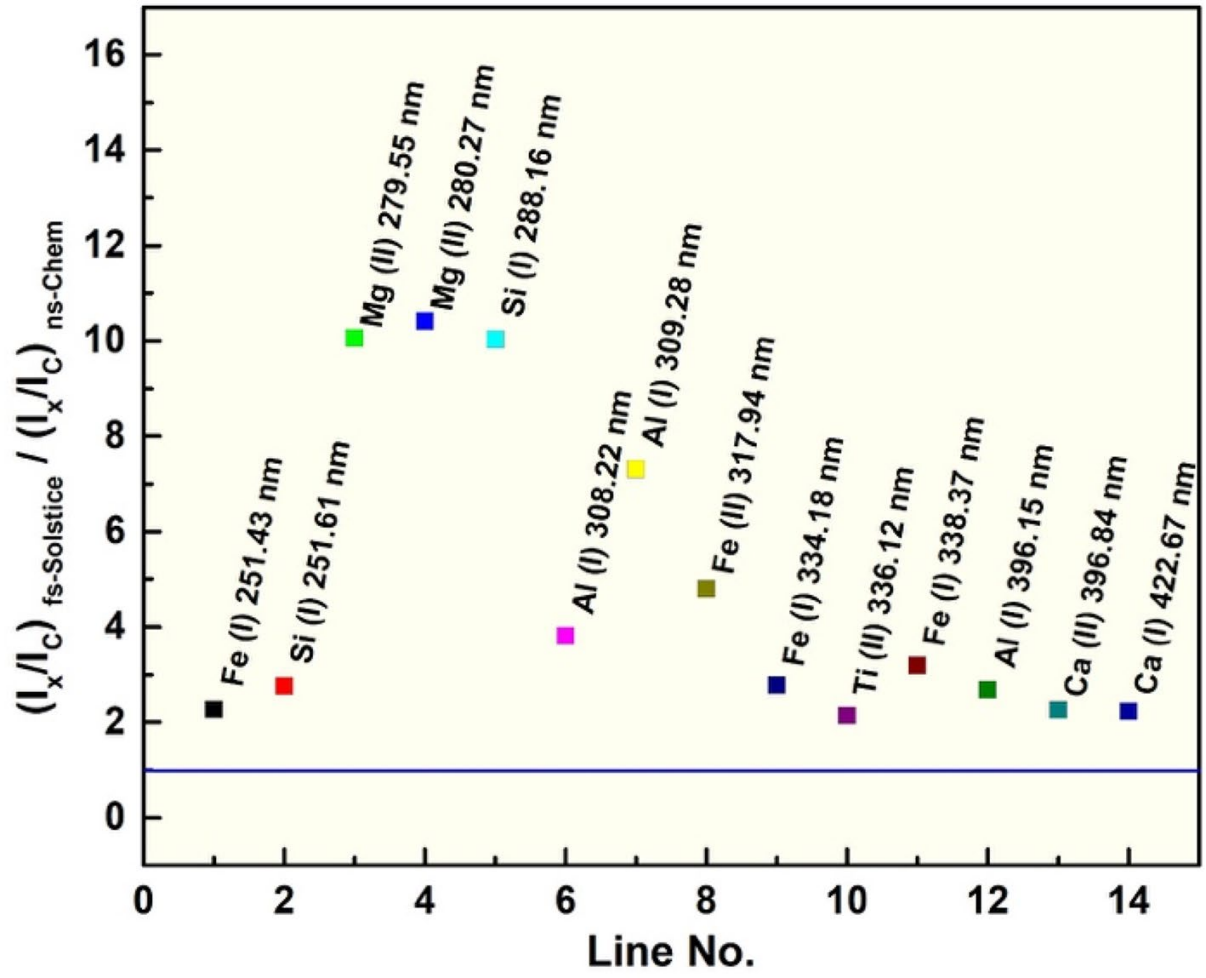
Relative sensitivity factors for 6 mineral elements: Fe, Si, Mg, Al, Ti, and Ca in coal matrix vary between 2 to 10 showing enhanced sensitivity of *fs-Solstice* system relative *ns-Chem* system for minerals detection.

Figure 4 depicts the mean RSFs for Fe, Si, Mg, Al, Ti, and Ca elements in 40 coal samples and are higher than 1 for all elements. RSFs vary between 2 and 10, showing enhanced detection sensitivity for *fs-Solstice* system relative to its ns counterpart. For prominent lines such as Mg II 279.55 nm, Mg II 280.27 nm (commonly used for plasma temperature correction to cancel its dependence on line intensities^46^), and Si I 288.16 nm (commonly used as internal standard), RSFs are ~ 10 indicating 10 times enhanced detection of those lines in a normalized spectra to its dominant element, i.e. carbon to by *fs-Solstice* system. Meanwhile, the response factor for Fe, Al, Ti, and Ca elements shows RSFs between 2 and 7. Gross et al.^47^ correlated the RSFs of 4 alkali metals in single-particle detection to both the ionization potential and lattice energy. A matter of fact is that for multicomponent analysis, factors such as the atomization/ionization energy, lattice energy, absorption cross-section at the ionization wavelength, and laser-particles interaction—all vary within each plasma and contribute to the final discernment in each system. Zhang et al.^12^ demonstrated that the discrepancy of RSFs between fs and ns LAs is attributed to the fundamental basis of high-irradiance laser ionization process in case of fs ablation. The RSFs variations reduced to half for the fs system relative to ns-laser mode indicating reduction in matrix-effects. Owing to the ultra-intense laser irradiance of fs laser ablation, atoms in lattice are atomized and ionized through multiphoton ionization, tunnel ionization, electron impact ionization, and avalanche ionization leading to a metastable plasma cloud with direct ejection of ions in the *solid–gas-mixture* phase^48^. The higher RSFs for elemental detection makes *fs-Solstice* system a competitive choice for industrial coal quality analysis applications, and indeed, for monitoring environmental pollutants of coal combustion reactions. Moreover, significantly high response factors for mineral elements adds a one-point advantage for *fs-Solstice* system far beyond *ns-Chem* system for the detection of minor and trace elements compared to x-ray diffraction (XRD) which provides little information on trace elements associated with coal minerals.

**Figure 5:**
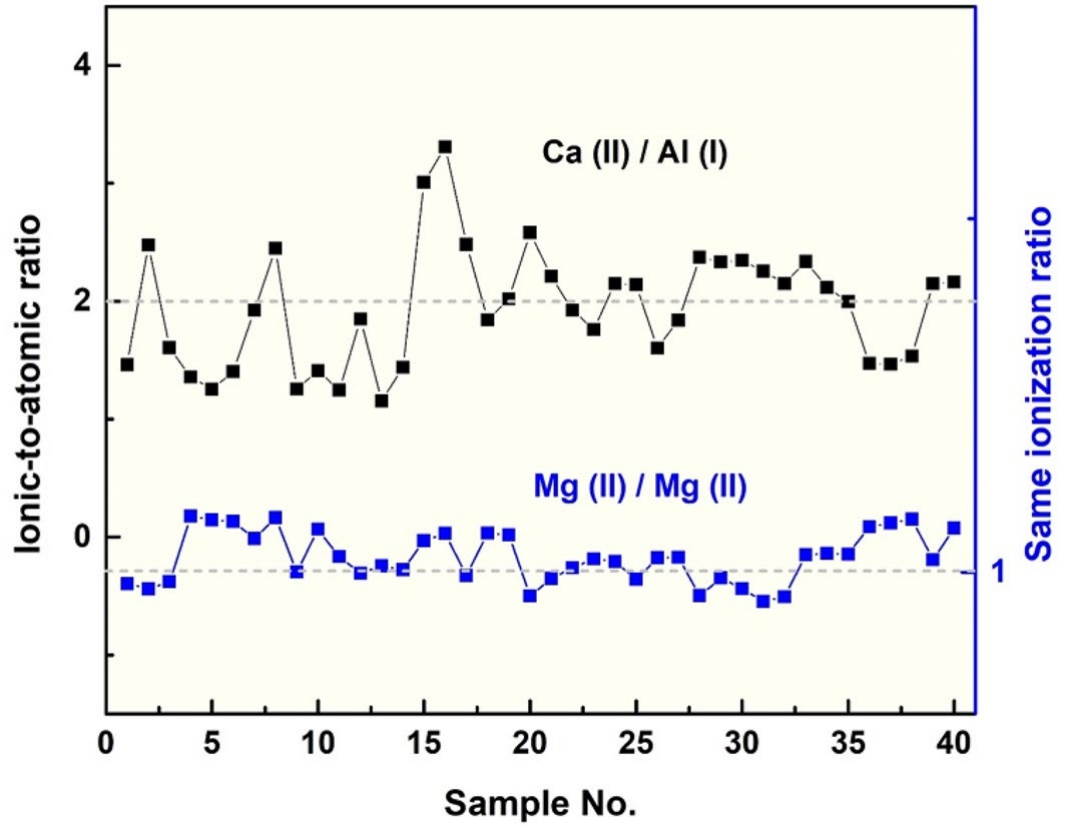
Relative sensitivity factors of ionic-to-atomic and ionic-to-ionic lines detected within *fs-Solstice* spectra proving abundant presence of ionic lines in fs plasmas.

The RSFs of ionic-to-atomic and ionic-to-ionic lines within *fs-Solstice* spectra are further studied to investigate the abundance of ionic lines in fs plasma and their persistence in different spectra (40 spectra correlating to 40 samples in use)—see Fig. 5. The RSFs of Ca II 393.36 nm to Al I 394.40 nm lines show variations around 2 with average RSF of ~ 2 ± 0.5 for all samples. This indicates enhanced presence/abundance of ionic lines in fs plasmas. While, the average RSF of Mg II 280.27 nm to Mg II 279.55 nm has a mean value of ~ 1.02 ± 0.04 showing similar presence for the same transition states. It is important to note that both of the selected transition sets are closely spaced to avoid the detector sensitivity issues for further apart transitions in the optical spectrum. The plentiful population of both atomic and ionic lines in fs coal plasma is in contrast to several research work, in which dominancy of atomic lines was reported. Freeman et al.^39^ demonstrated neutral emission with *little-to-no* ionic emission from fs-plasma of brass sample in both vacuum and atmospheric conditions. LaHaye et al.^40^ claimed fs-LA plume to be more atomic due to absence of laser-plasma coupling relative to the more ionized plume of ns-LA, where laser interacts with plasma forming higher early excitation temperature and electron density. Herein, the results show the prominence of higher ionic states in fs plasmas despite shorter life time and cooler plasmas due to the fundamental ablation mechanisms. In fs-LA, the fast dissipation of pulse energy density into the sample with minimized thermalization incur Coulombic regime of plasma hydrodynamics where electrostatic ejection and formation of atomic, ionic, and molecular fragments is dominant. The high ionic states abundance is found to increase with laser power density^49^. In ns-LA, the thermalization and plasma heating results in lower ionization states^50^ and higher plasma parameters (both higher plasma temperature and electron density) due to the dissipation mechanism of thermal energy into sample. The contrarieties in laser energy coupling between fs and ns-LAs are clarified by craters morphologies.

### Craters topology

The ablation features; i.e. mass and yield, of both *fs-Solstice* and *ns-Chem* systems are investigated by focused ion beam scanning electron microscopy (FIB-SEM) and coherence scanning interferometry (CSI) to better evaluate the formation mechanism of coal ejecta induced by fs-LIBS system relative to that obtained by the ns-LIBS system.

#### FIB-SEM images

**Figure 6:**
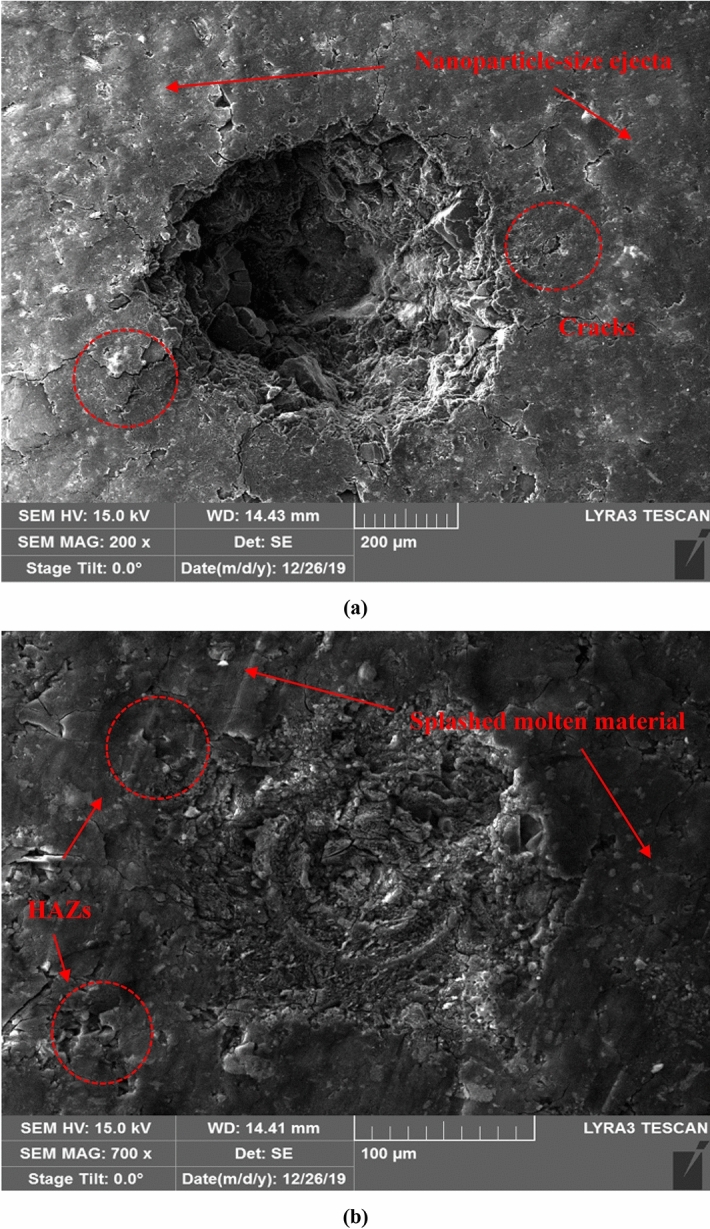
Focused ion beam scanning electron microscopy (FIB-SEM) images of laser ablation craters formed using (**a**) *fs-Solstice* sytem where: no rim not heat affected zones (HAZs), cracks at the crater’s edge due to strong fs laser hit, and powdery appearance of ejecta are observed; (**b**) *ns-Chem* system where: rim, HAZs, molten and splashed particles are observed.

Figure 6a elucidates a precise crater with Gaussian-like profile obtained via *fs-Solstice* system ablation, while the crater bottom can be clearly seen. The sample surface shows no rim formed nor heat-affected zones (HAZs) of molten material. In contrast, both rim and HAZs are observed in case of *ns-Chem* ablation, see Fig. 6b. Cracks at the crater’s edge are observed due to a strong fs-laser hit (a pulse train of 100 signals) in ablation with electrostatic nature which are not observed in case of *ns-Chem* ablation (single shot) and replaced with scattered HAZs of molten material formed in thermal explosion reaction. Splashed particles ejected from molten ablated mass are distinguished on coal sample after ns-LA, and according to their temperature at the landing time over the surface, they appear as HAZs or large agglomerates. For fs-LA, powdery appearance of nanoparticles spread around the crater indicates that the coal ejecta is ablated in nanoscale particle size. This is in agreement with the report by Liu et al.^51^ who studied the ablated aerosol vapor collected on silicon substrate after fs and ns-LAs of brass alloy. SEM images showed ejecta formed by fs-LA were ~ 100 nm while those formed by ns-LA were in 100 s nm to μm size scale. The slow thermalization and re-solidification of the ablated matter on sample’s surface are significant sources of matrix effects and fractional evaporation. It should be noted that *ns-Chem* is in optimal operation conditions of minimized HAZ to reduce matrix effects and to enhance signals repeatability. The *fs-Solstice* system, which is not exactly working in optimal operation conditions for coal analysis, shows reduction of thermal effects and proves absence of fractional evaporation.

#### CSI profiles

**Figure 7:**
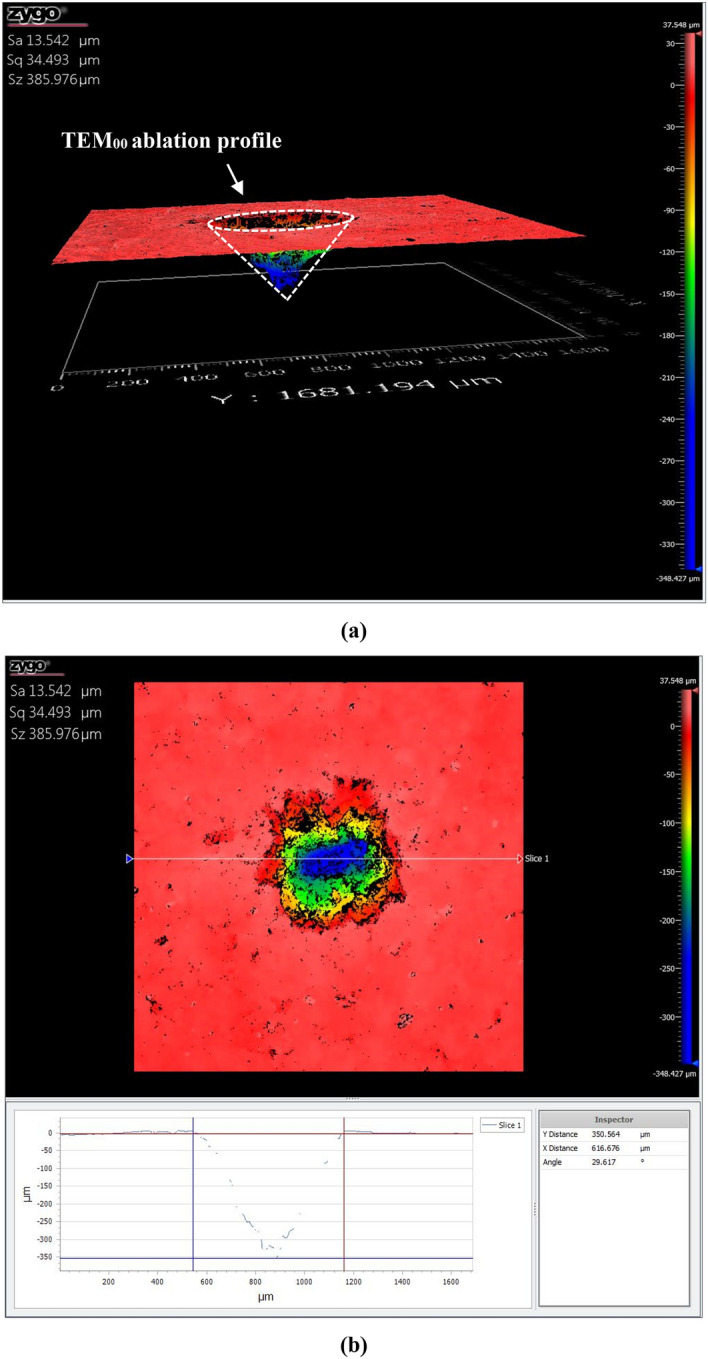
3D surface profilometer images of coal sample after ablation by *fs-Solstice* system: (**a**) is the 3D profile and (**b**) is surface profile view. The ablated volume has a circular-cone structure accompanied with absence of molten rims and heat affected zones. The crater diameter is 617 μm and its depth is 350 μm.

**Figure 8:**
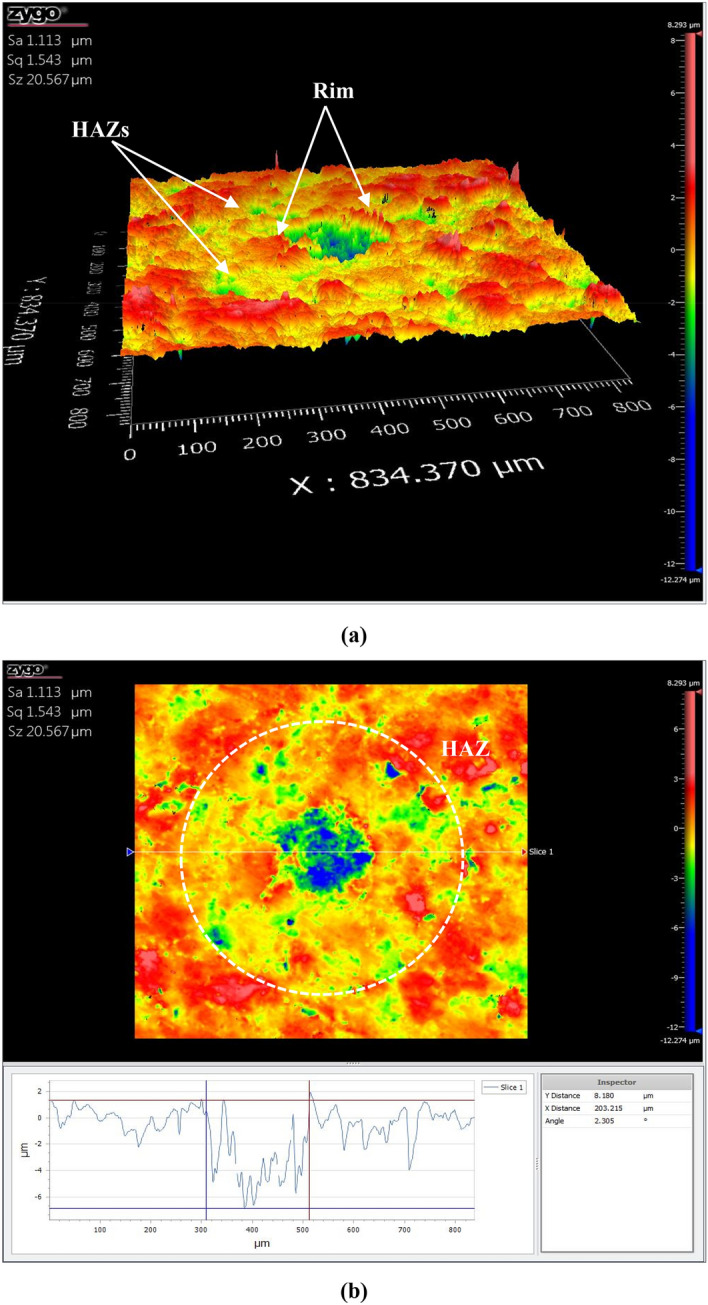
3D surface profilometer of coal sample after ablation by *ns-Chem* system: (**a**) is the 3D profile and (**b**) is surface profile view. The ablated volume can be simulated as a truncate-cone structure with clear thermal effects; rims and heat affected zones (HAZs). The crater diameter is 203 μm and its depth is 8 μm.

The 3D surface profilometer images show the alteration occurred to the coal sample after laser ablation. Figure 7 shows the Gaussian beam profile of *fs-Solstice* laser which yields a cone-shaped crater accompanied with absence of molten rims and HAZs. The fs-crater has a surface diameter of 617 μm and depth of 350 μm. For *ns-Chem* system, rims as molten heights with pointed tips (look like stalactites) are formed around the crater with HAZs are shown as random thermal grooves/modifications of coal surface, see Fig. 8. The ns-crater has a surface diameter of 203 μm and depth of 8 μm. While *fs-Solstice* system employs a fs-laser with Gaussian beam profile, *ns-Chem* system has a laser source with top-hat beam profile. Therefore, assuming the ablated masses have right structures, *fs-Solstice* yielded ~ 3.5E−05 cm^3^ ablation volume (considering the fs-crater has circular cone structure) and *ns-Chem* ablated ~ 1.5E−07 cm^3^ (considering the ns-crater has truncated cone structure). Using the bituminous coal density (1.346 g cm^−3^^52^), the ablated masses are ~ 40 and ~ 0.2 μg for *fs-Solstice* and *ns-Chem* systems, respectively. The ablation yield is defined as the mass ablated per laser pulse which gives 0.4 μg/pulse for fs-Solstice system (a pulse train of 100 signals) and 0.2 μg/pulse for *ns-Chem* system. The energy densities responsible for this ablation yield are calculated from the incident laser energies and spot sizes for each system. For *fs-Solstice* system, the energy density is: (5.7 × 100 × 10^–3^) J/(π × (617 × 10^–4^)^2^ cm^2^ = 47.66 J cm^−2^. For *ns-Chem* system, the energy density is: (90 × 10^–3^) J/(π × (203 × 10^–4^)^2^ cm^2^ = 69.51 J cm^−2^. This means that *fs-Solstice* system has double the ablation yield of *ns-Chem* system and ablates a mass which is 200 times more that ablated by *ns-Chem* system with ~ 2/3 of the energy density employed. Table 5 summarizes the ablation features for the evaluation of *fs-Solstice* relative to *ns-Chem*.

**Table 5:** Craters and ablation features of *fs-Solstice* and *ns-Chem* systems for coal samples analysis.

	*fs-Solstice*	*ns-Chem*
Crater width/μm	617	203
Crater depth/μm	350	8
Crater volume/cm^3^	3.5 × 10^–5^	1.5 × 10^–7^
Mass of ejecta/μg	40	0.2
Ablation yield/μg per pulse	0.4	0.2
Energy density/J cm^−2^	47.66	69.51

Several contributing factors have been discussed in literature to explain the phenomenology of enhanced energy density deposition in fs laser matter interactions, including:

1. *Enhanced laser energy coupling* Considerably contrastive physics of energy deposition between fs and ns-LA regimes is attributed to the pulse duration; i.e. time scales in which intense laser energy deposits. The fs laser pulse has a pulse duration τ_L/fs_ shorter than the electron-lattice relaxation time τ_e_. Therefore, fs-pulse energy couples to electron carriers which follow Drude hydro-dynamics with various scattering and collision events to relax energy into lattice. Hot electrons cause a high-pressure buildup within the crystal which is then released via mechanical expansion in electrostatic ablation. The extremely rapid isochoric heating of lattice pushes the system into metastable state where homogenous nucleation of gas bubbles in solid–vapor phase explosion takes place. Moreover, mechanical fragmentation and spinodal decomposition are two effective ablation mechanisms happening at high energy density regime. The two-temperature model^53,54^ is used to describe the energy relaxation between electrons and lattice, as:
  3 $${C}_{e}\frac{\partial {T}_{e}}{\partial t}= -\frac{\partial \left[-{k}_{e}\frac{{\partial T}_{e}}{\partial z}\right]}{\partial z}- \gamma \left({T}_{e}-{T}_{i}\right)+{I}_{a} \alpha \mathrm{exp}(-\alpha z )$$
  4 $${C}_{i}\frac{\partial {T}_{i}}{\partial t}= \gamma ({T}_{e}-{T}_{i})$$
  where; ${C}_{e}$ is the heat capacity per unit volume of the electron subsystem, ${T}_{e}$ is electron temperature, $Q\left(z\right)$ is the heat flux which is a function at z direction perpendicular to the target surface and given by: $Q\left(z\right)= - {k}_{e}\left(\frac{\partial {T}_{e}}{\partial z}\right)$, $\gamma$ is the electron-lattice coupling parameter, ${T}_{i}$ is lattice temperature, $S$ is the laser heating source function and given by: $S=A \alpha I\left(t\right)\mathrm{exp}-\alpha z$, ${C}_{i}$ is the heat capacity per unit volume of the lattice subsystem, ${k}_{e}$ is the electron thermal conductivity, $A$ = 1 − *R* is the surface transmittivity, $\alpha$ is the material absorption coefficient, and ${I}_{a}$ is the absorbed laser intensity. For a fs-pulse, τ_L/fs_ << τ_e_, and hence, $({C}_{e} {T}_{e})/{}_{\mathrm{L}/\mathrm{fs}})\gg \gamma {T}_{e}$ and D_e_ τ_L/fs_ < ${\alpha }^{-2}$ where D_e_ is the electron thermal diffusivity. The threshold fluence for fs-evaporation is given by: ${F}_{th/fs}\approx \rho\Omega ({D}_{e} {}_{L/fs}$)^1/2^. If the electron heat conduction is neglected, then the fs-plasma expansion is adiabatic, ultrafast, and non-thermal ablation where threshold fluence is minimized with pulse width. Figure 9 shows 2D CSI profiles of a fs-single shot (not a pulse train) and a ns-single shot of a coal sample. The reduced thermal effect and enhanced laser energy deposition are clearly seen for *fs-Solstice* pulse relative to *ns-Chem* one.

**Figure 9:**
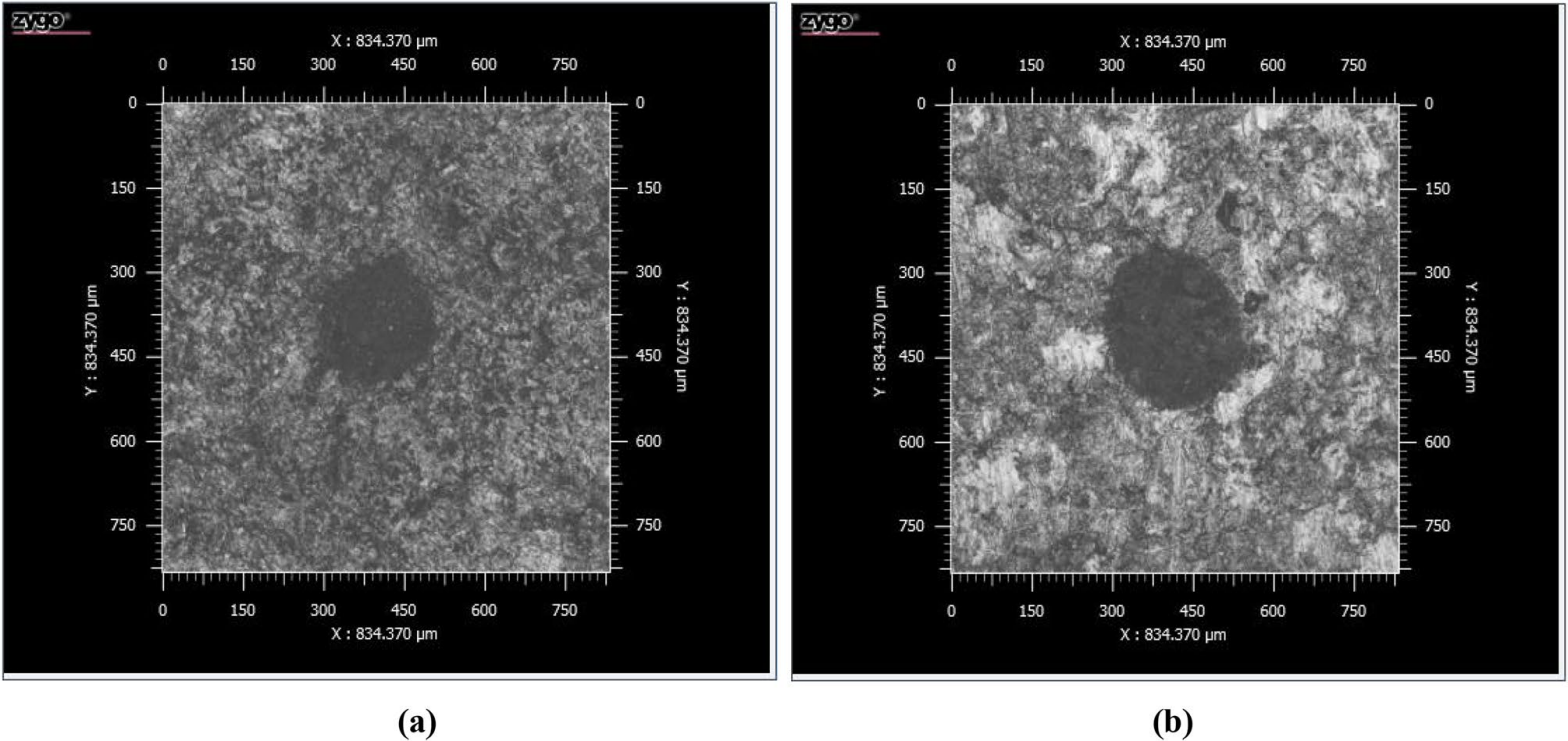
2D surface profilometry images of single shots ablation using *fs-Solstice* system in (**a**) and *ns-Chem* system in (**b**) clarifying the reduced thermal effects of fs-LA.
2. *Enhanced residual thermal energy deposition* At high fluences and prior to the hydrodynamic expansion of ablated ejecta, direct ionization of ambient gas (collisional environment) can occur due to highly energetic electron-carriers with fs-pulse energy directly deposited into them. A high-pressure plasma layer is generated at the interaction zone which is believed^55–57^ to enhance electrons-lattice thermal coupling by retaining (or confining) coupling process at a time interval required for rarefaction wave (decreased density and pressure, molecules stretch time) to travel from plasma periphery to center of irradiated spot and then enhance the re-deposition of ablated ejecta—see Fig. 10.

**Figure 10:**
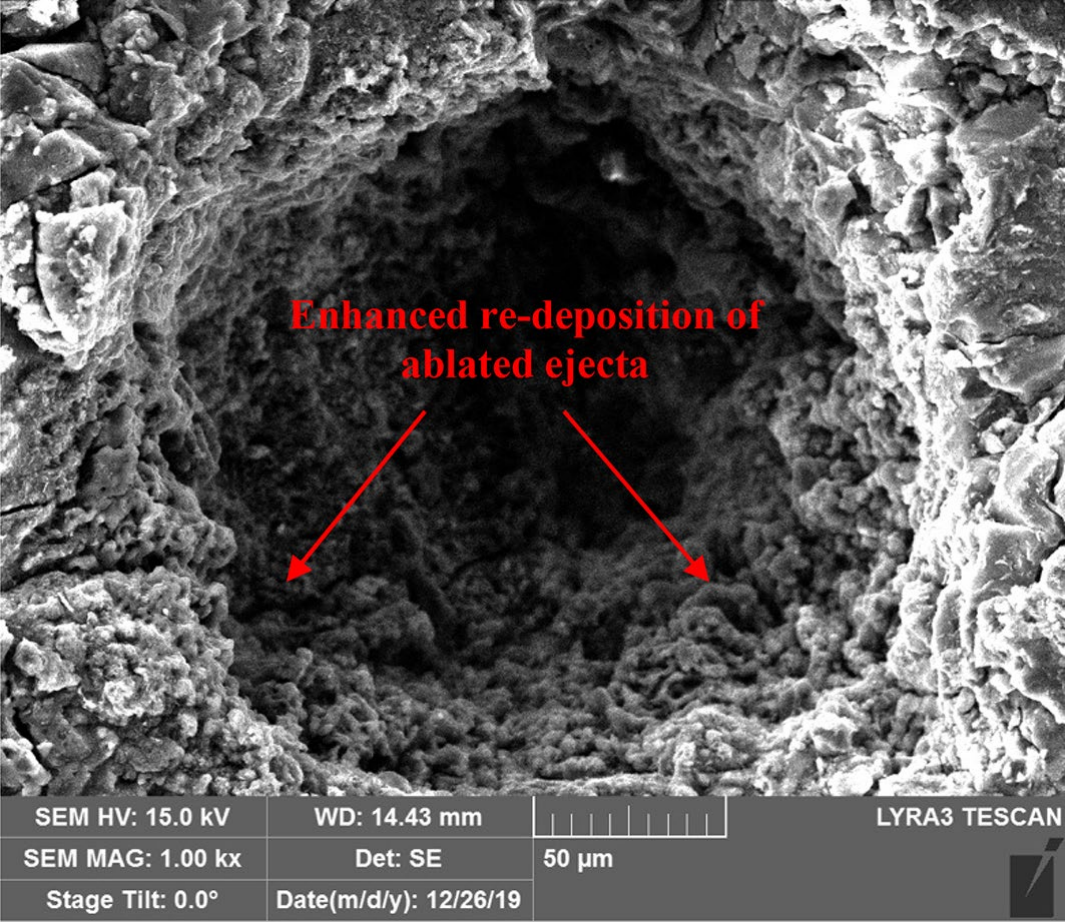
Focused ion beam scanning electron microscopy (FIB-SEM) image of the interior part of coal crater ablated by *fs-Solstice* system (a train pulse of 100 signals). The re-deposited ablated ejecta inside the crater resemble those formed on the surface as nanoparticle-sized which confirms the enhanced thermal energy deposition for fs-LA.
3. *Reduced threshold fluence of ablation in fs-train pulse* Incubation phenomena^58,59^ is behind the reduced threshold fluence of ablation in a fs-train pulse. Physical ansatzes are developed to link the incubation to the formation of laser-induced states formed due to the storage cycle of thermal stress–strain energy (material fatigue) and the creation of laser induced defects, ripples, and nano-cracking^60–62^. Meanwhile, surface reflectivity was observed to decrease in fs train pulse^63,64^. The Nth declined fluence is reported as: ${F}_{th/fs}^{N}\approx {F}_{th/fs }^{1}{N}^{-\xi }$ where ${F}_{th/fs }^{1}$ is the single-pulse threshold fluence and $\xi$ is the incubation coefficient. For a beam with a Gaussian beam profile, the diameter of the ablated crater is related to applied fluence^65^: ${D}^{2}=2 {\omega }_{o}^{2}\mathrm{ln}(F/{F}_{th})$; where ${\omega }_{o}$ is 1/e^2^ radius of laser light distribution and $D$ is the formed crater diameter. The threshold fluence at the 100th pulse in the train pulse is then deduced: ${F}_{th/fs}^{100}$ ~ 0.102 J cm^−2^ ($D$ = 617 μm and ${\omega }_{o}$ = 176 μm).

### Plasma parameters

Spectral lines characteristics (intensities, resolution, widths, and repeatability) and crater morphologies (depth, width, and precision) are causally connected to the matter physicochemical structure and laser-matter interaction properties; i.e. plasma parameters. Hence, plasma parameters (plasma temperature and electron number density) are utilized as an evaluation measure to see the parametric plasma conditions led to the corresponding spectral features, abundant species detected in plasmas, and craters topology by *fs-Solstice* system relative to those generated by *ns-Chem* one. Plasma temperatures are extrapolated from the electronic temperature of plasma components assuming a thin plasma in local thermodynamic equilibrium according to Griem’s criterion^66^. Boltzman’s law^67^ is applied for four closely-spaced Mg II lines to deduce plasma temperature as:

5 $$\mathrm{ln}\left(\frac{{\lambda }_{ki}{I}_{ki}}{{g}_{k}{A}_{ki}}\right)= -\left(\frac{{E}_{k}}{{k}_{B}T}\right)+C$$

where; ${\lambda }_{ki}$, ${I}_{ki}$, ${g}_{k}$, ${A}_{ki}$, ${E}_{k}$, ${k}_{B}$, and $C$ are emission wavelength, integral intensity of emission line, degeneracy of upper level energy, transition probability, upper-level energy, Boltzmann constant, and a constant, respectively. Plasma temperatures are obtained using slope of the fitted line. Non-resonant ionic Mg II lines: 279.10, 279.56, 279.81, and 280.27 nm are selected to deduce the temperature. Our choice is based on using lines with same ionization states and closely-spaced to reduce the effect of instrumental efficiency bias. For each sample, 100 spectra are averaged and normalized to the whole spectral area then Mg II lines are used to deduce the sample’s plasma temperature. The Mg II lines exhibit ~ 20 times higher normalized peak intensities in case of *fs-Solstice* spectra relative to *ns-Chem* spectra. This is attributed to the accumulation over fs train pulses, higher resolution, and detector sensitivity of the spectrometer used in the fs system. Nevertheless, the integrated line intensities of Mg II lines are used without background subtraction to encounter for population-averages of the local electronic temperature through the lifelong detection window for each system. Table 6 collects the spectral line parameters from NIST database^20^.

**Table 6:** List of Mg II lines for plasma temperature calculation and corresponding spectroscopic parameters.

λ/nm	E_k_/eV	E_i_/eV	A_ki_/s^−1^	g_k_	g_i_
279.10	8.86	4.42	1.63 × 10^–3^	6	2
279.56	4.43	0.00	2.60 × 10^8^	2	4
279.81	8.86	4.43	4.79 × 10^8^	4	6
280.27	4.42	0.00	2.57 × 10^8^	2	2

The electron density is determined by using the full-width at half maximum (FWHM) of spectral lines according to Stark broadening^68^:

6 $${N}_{e}=[\Delta {\lambda }_{Stark}\times \frac{{10}^{16}}{2\omega }]$$

where; $\omega$ is electron impact parameter, and $\Delta {\lambda }_{Stark}$ is FWHM of Stark broadened profile. Lines’ profiles are affected by different broadening mechanisms. Stark broadening and instrumental broadening are accounted for in laser-induced plasmas; considering contribution of other broadening mechanisms is ignorable. The C I transition at 247.86 nm is used for electron density measurements and is fitted using a Voigt profile which considers Stark broadening by Lorentzian profile and instrumental broadening by Gaussian profile. Stark broadening is de-convoluted after inserting instrumental broadening for each system (22 pm and 0.5 nm FWHM for *fs-Solstice* and *ns-Chem* systems respectively measured using a low-pressure mercury lamp) as follows:

7 $$\Delta {\lambda }_{measured (Voigt) }= {\left[{\left(\frac{\Delta {\lambda }_{Stark \left(Lorentz\right)}}{2}\right)}^{2}+{\left(\Delta {\lambda }_{Instru. \left(Gauss\right)}\right)}^{2}\right]}^\frac{1}{2}+(\frac{\Delta {\lambda }_{Stark \left(Lorentz\right)}}{2})$$

**Figure 11:**
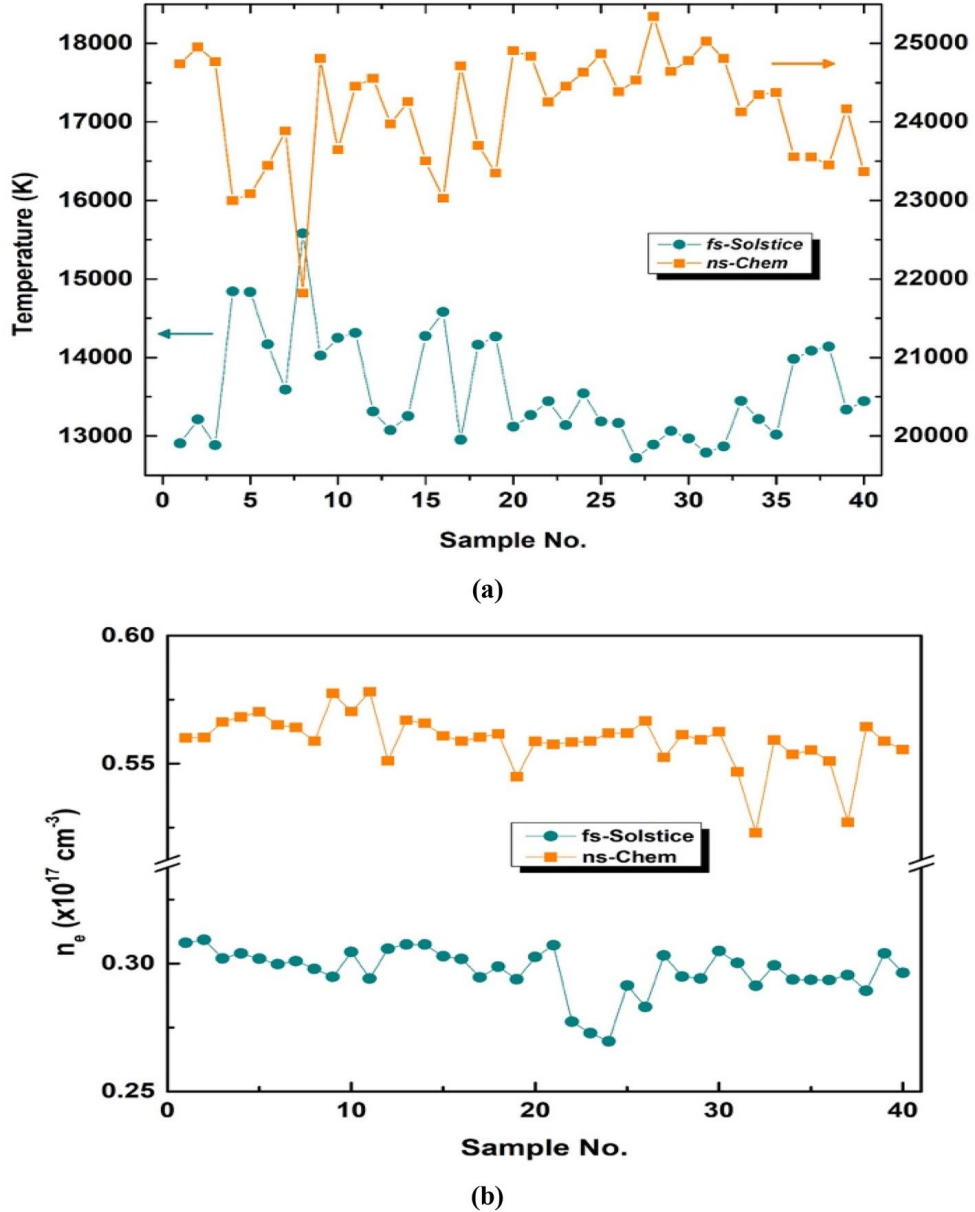
Plasma temperatures in (**a**) and electron densities in (**b**) for 40 coal samples ablated with *fs-Solstice* and *ns-Chem* plasmas. The average temperature value for fs system is 13,583 ± 679 K while for ns system is 24,151 ± 732 K. The average electron number densities are 2.97 ± 0.09 × 10^16^ cm^−3^ for fs plasma and 5.59 ± 0.10 × 10^16^ cm^−3^ for ns plasma.

Figure 11 depicts the plasma temperatures (in a) and electron densities (in b) for 40 coal samples analyzed by each system. The average plasma temperature of all samples analyzed by *fs-Solstice* system is 13,583 ± 679 K. While, it is 24,151 ± 732 K for *ns-Chem* system. Similarly, the average electron density is 2.97 ± 0.09 × 10^16^ cm^−3^ for *fs-Solstice* and is 5.59 ± 0.10 × 10^16^ cm^−3^ for *ns-Chem*. FWHM of other minor elemental lines (not shown) expressed alike trend suggesting denser plasmas obtained by *ns-Chem* system ablation parameters. One might consider that hotter and denser plasma conditions are better to obtain more ablation ejecta and spectral information. While, *fs-Solstice* evaluation results of the performance measures: RSFs and craters topology with plasma parameters show that cooler and less dense plasma may form less chaotic conditions for different elemental species to evolve and gentle craters to form. The laser-plasma interaction is virtually absent, and thereby, fs-plasmas are overcoming the shielding and re-heating effects typically caused by ns-laser excitation^33^. Therefore, *fs-Solstice* plasmas have lower plasma temperatures and less electron density values relative to *ns-Chem* plasmas. From physics point of view, as soon as the formed plasma temperature in the interaction zone reaches energy magnitudes enough for breaking/melting, vaporization, and ionization of matter species, it validates for spectroscopy elemental detection. In a sense, an *ideal* plasma performs stoichiometric ablation with least chaotic behavior. The electrostatic ablation nature and the absence of laser-plasma interaction promote *fs-Solstice* plasma as competitive source for non-stochastic plasma parameters.

### Measurement-to-measurement repeatability

**Figure 12:**
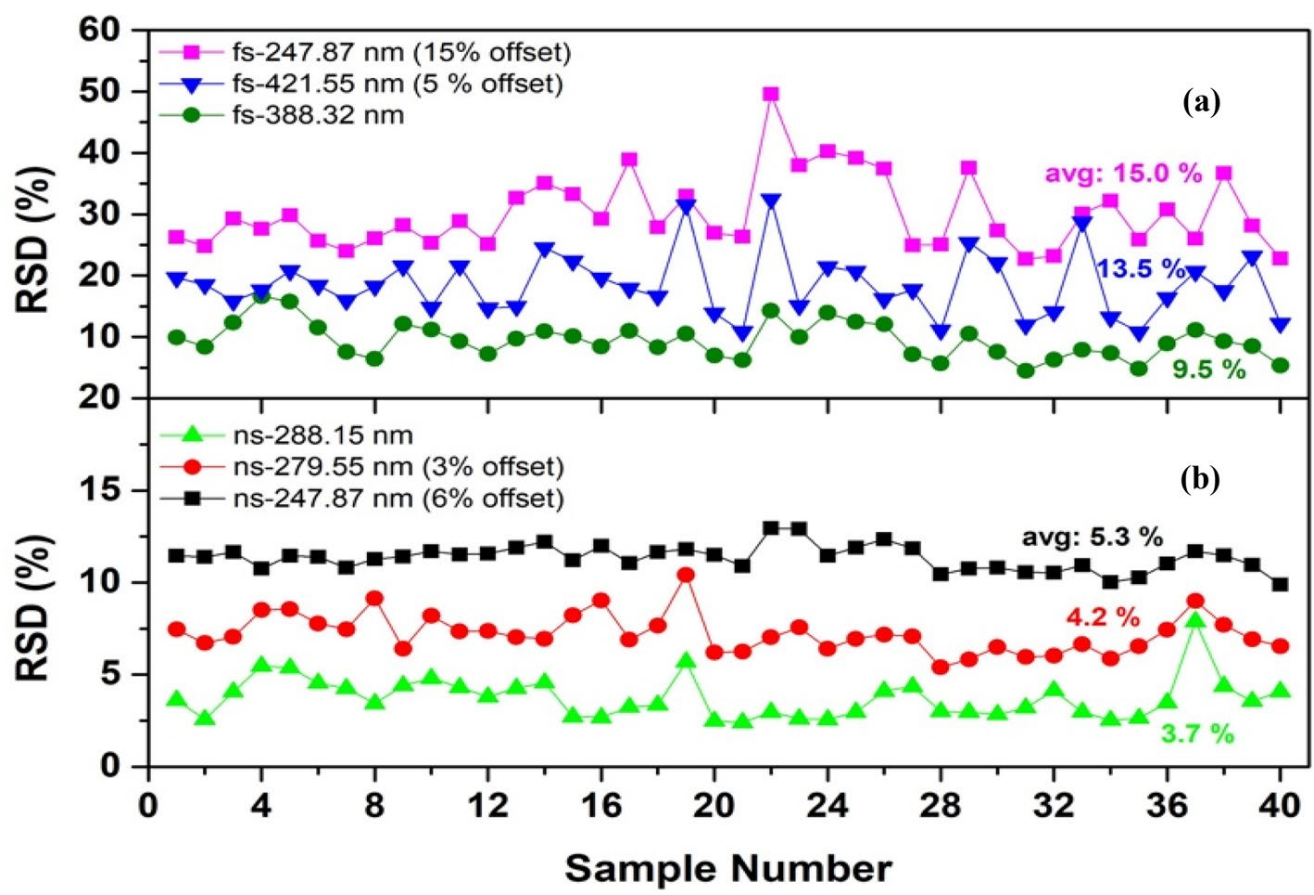
Relative standard deviations (RSDs) of 100 spectra for the 40 coal samples ablated by: (**a**) *fs-Solstice*, and (**b**) *ns-Chem* systems with average RSDs of 9.7% and 2.5% for the two systems respectively. Offsets are to avoid points’ overlapping for clarity.

Spectral repeatability is accessed by average measurement-to-measurement RSDs of raw spectral lines. Figure 12 shows RSDs of 100 measurements (ablation matrix of 10 × 10 positions) for 40 coal samples ablated by *fs-Solstice* (in a) and *ns-Chem* (in b) systems. The average RSD of C I 247.87 nm line for all samples is 15% for *fs-Solstice* spectra; ~ 3 times higher than that obtained by *ns-Chem* spectra (average RSD of 5.3%). The Sr II 421.55 nm line and the molecular band CN at 388.32 nm show average RSDs of 13.5% and 9.5% respectively for *fs-Solstice* spectra. While, other lines including Si I line at 288.15 nm and Mg II at 279.55 nm show lower average RSDs of 3.7% and 4.2% respectively in case of *ns-Chem* spectra. To get an overall estimation of each system’s repeatability, the 100 lines with highest repeatability obtained by each system are investigated. For *fs-Solstice* system, 100-highest repeatability lines have average RSD of 9.7%. The repeatable lines are branches of CN or C_2_ bands due the stable formation of molecules; i.e. fragmentation. For *ns-Chem* spectra, 100-highest repeatability lines have average RSD of 2.5%. The repeatable lines are atomic/ionic lines of mineral elements.

The fact that *fs-Solstice* system has: stable crater morphology, ionized plasma cloud which is not interacting with the laser tail, and ejecta formed in a cool plasma with considerably low chaotic behavior in a short lifetime, encounter the expectation of higher signals repeatability relative to *ns-Chem* system. However, *ns-Chem* system is optimized for best-signals repeatability and precise quantitative analysis as an offline coal analyzer already operating in power plants. The work on the direction of optimizing the *fs-Solstice* system repeatability for coal analysis would uncover interesting aspects about plasma modeling based on physical background where accuracy is significantly boosted.

### Industrial scale-up of fs-LIBS: applicability and problems

Ultrafast lasers are unique by incredibly high peak intensities and interaction timescale faster than the lattice disorder and heat diffusion. Since their development over half a century, researchers continue to uncover new phenomena about laser-matter interactions. As for spectroscopic techniques, advances provided by femtosecond lasers have been directed to solve different analytical tasks. LIBS benefits from femtosecond lasers and the review paper by Labutin et al.^69^ foresaw a brighter future for numerous fs-LIBS applications due to state-of-the-art findings reviewed in studies. Howbeit, a slow mainstream adoption of fs- LIBS is observed relative to the analytical and industrial problems the technique is capable to solve. This is attributed to: (1) Unavailability of reliable/stable *high energy* femtosecond lasers; (2) Lack of fs-physics understanding quadrated with analytical capabilities; (3) Commercial restraints such as high cost, maintenance, and performance; (4) Presence of other affordable techniques. In fact, the first review paper of fs-LIBS by Gurevich et al.^70^ outlined two important points about scaling up fs-LIBS technique. The first point is that a replacement of current widely-used technique cannot be expected without a better performance in terms of analytical figures-of-merit by fs-LIBS based on understood plasma formation mechanisms. The second point is that a booming growth of ongoing trends such as femtosecond micromachining and surgery applications can provide a way for simplified, compact, and all-solid-state femtosecond lasers. This means that: (1) Ablation physics plays a significant rule in understanding the analytical capabilities and limitations of fs-LIBS. Without such detailed physics understanding, effective optimization of fs-LIBS instrumentation is hardly possible; (2) Industrial applications of fs-LIBS and trendy applications (e.g. thin films deposition, quantum dots synthesis, chemical imaging, standoff environmental sensing, etc.) where fs-LIBS performs inline operation monitoring are gateways for effective commercialization where cost and size reduce significantly. At present, some research groups around the world are focusing on both directions of analytical performance of fs-LIBS quadrated to its ablation physics and industrially-related applications. We believe that for coal analysis industry with a huge market size, operation efficiency and accuracy have decisive impacts on a technique’s utilization and fs-LIBS introductory-study presented in this letter is a challenging new task for a wide research direction of *in-field* adaption of the technique. Table 7 summarizes the performance measures and evaluation results of *fs-Solstice* system under assessment relative to *ns-Chem* system.

**Table 7:** Performance measures and evaluation results of *fs-Solstice* system relative to *ns-Chem* for coal analysis.

Performance measure	*fs-Solstice*	*ns-Chem*	Evaluation
Correlation coefficients	***r*** $\ge$ 0.949 Different lines with most identified within molecular bands	***r*** $\le$ 0.923 Same lines correlated to carbon content and heat value	Higher representativeness and selectiveness of *fs-Solstice* spectra to coal property Formation of molecular fragments correlated to parent molecules due to fs-laser ablation nature
Relative sensitivity factor	RSFs ≈ 2–10		Enhanced detection sensitivity of ionic and atomic species by *fs-Solstice* system
Craters topology	No rim nor heat-affected zone Ejecta in nanoparticle-size Ablated mass = ~ 40 μg by 47.66 J cm^−2^	Clear rim and heat-affected zone Splashed and molten material Ablated mass = ~ 0.2 μg by 69.51 J cm^−2^	Higher ablation yield of *fs-Solstice* system (0.4 μg/pulse relative to 0.2 μg/pulse for *ns-Chem* system) due to enhanced laser-energy coupling
Plasma parameters	Plasma temperature = 13,583 ± 679 K Electron density = 2.97 ± 0.09 × 10^16^ cm^−3^	Plasma temperature = 24,151 ± 732 K Electron density** = **5.59 ± 0.10 × 10^16^ cm^−3^	Cooler and less-dense fs-plasma incorporate less chaotic behavior for stable ablation
Repeatability	RSDs = 9.5–15%	RSDs = 3.7–5.3%	Optimization of *fs-Solstice* system repeatability is needed

## Conclusion

Femtosecond lasers provide enhanced analytical capabilities for different LIBS applications. In this work, we have conducted an evaluation study of a one-box femtosecond laser system for the application of coal property analysis relative to an industrially-applied coal analyzer. The evaluation study is based on using 5 different objectives/measures: spectral correlations, relative sensitivity factors, craters topology, plasma parameters, and repeatability—which cover different analytical performance aspects. The fs-LIBS system reveals competitive results to those obtained by the reference coal analyzer:

- Higher representativeness (correlation coefficients) of fs-spectra to coal properties with different lines correlated to each coal property (higher selectiveness).
- Enhanced detection sensitivity and presence/abundance of ionic lines in fs-plasmas with relative sensitivity factors of 2–10.
- Precise ablation craters with no rim nor heat-affected zones of splashed/molten material due to enhanced laser energy coupling for fs-plasmas. The fs-system has higher ablation yield of 0.4 μg/pulse vs. 0.2 μg/pulse for the reference system.
- Cooler and less-dense fs-plasmas incorporate less chaotic behavior for stable ablation.
- Further studies have to be conducted for *in-field* optimization of fs-system, includes: signals repeatability for precise quantitative results, operation under harsh conditions, customization for different installation sets in power plants, etc.—the reference coal analyzer has higher signals’ repeatability.

## Materials and systems

### Samples

Samples used in this study were 40 standard powdery bituminous coal samples. Table 8 shows the certified chemical analysis of C, heat value, volatile, and ash contents. Air-dried coal pellets were pressed using a hydraulic tablet machine at 25-ton pressure for 3 min. Each pellet contained ~ 3 g powder coal and formed in dimensions of 30 mm diameter and 3 mm thickness after pressing. Two sets of samples were prepared under the same conditions, each to be analyzed by a system.

**Table 8:** Certified chemical composition of coal samples utilized in this study.

Sample code	Sample no.	Carbon (wt%)	Heat value (MJ kg^-1^)	Volatile (wt%)	Ash (wt%)
ZBM096	#1	81.45	31.86	11	10.09
ZBM098A	#2	78.58	32.5	31.68	8.25
ZBM099	#3	79.6	31.4	15.3	10.41
ZBM100A	#4	49.3	19.42	11.7	41.22
ZBM100B	#5	46.46	18.16	11.81	43.85
ZBM100C	#6	53.69	20.98	13.64	34.14
ZBM101	#7	67.08	26.85	18.5	22.8
ZBM102	#8	57.82	22.45	30.43	25.88
ZBM103A	#9	56.15	21.64	13.77	29.66
ZBM104	#10	53.63	21.33	13.5	33.56
ZBM105	#11	53.16	20.65	14	31.8
ZBM106	#12	72.45	29.44	30.31	13.68
ZBM108	#13	79.02	30.56	11.3	11.98
ZBM108A	#14	78.45	30.45	11.72	12.21
ZBM111A	#15	74.16	29.72	33.64	9.62
ZBM111B	#16	72.05	28.85	33.71	11.74
ZBM111C	#17	77.14	31.25	31.29	8
ZBM112	#18	59.65	23.72	28.5	25.17
ZBM112A	#19	61.2	24.35	28.96	23.5
ZBM113	#20	78.5	32.3	33.7	8.08
ZBM113A	#21	76.5	31.73	32.77	9.54
ZBM114	#22	74.6	29.5	33.3	6.97
ZBM115	#23	76.36	30.18	33.2	4.66
ZBM120	#24	77.28	30.57	31.74	6.38
ZBM121	#25	78.15	31.18	31.8	6.08
ZBM121A	#26	75.34	29.93	23.77	11.06
GBW11101G	#27	79.02	31.99	19.43	10.94
GBW11102X	#28	67.12	27.12	30.1	16.47
GBW11102Y	#29	69.64	28.4	33.32	13.61
GBW11107C	#30	78.16	31.89	28.42	8.88
GBW11107D	#31	75.12	30.57	33.07	9.01
GBW11108P	#32	66.92	27.01	30.01	16.26
GBW11108Q	#33	68.29	28.03	32.5	15.88
GBW11109N	#34	65.59	27.06	35.07	17.06
GBW11109O	#35	66.04	27.23	35.17	16.6
GBW11109Q	#36	53.66	22.03	29.28	31.01
GBW11110M	#37	44.99	17.84	17.13	42.14
GBW11110Q	#38	57.01	22.97	20.39	28.98
GBW11111O	#39	68.59	27.98	26.81	18.5
GBW11111P	#40	59.33	23.84	24.82	26.86

### Coal-analysis systems

#### Femtosecond-LA system (*fs-Solstice*)

**Figure 13:**
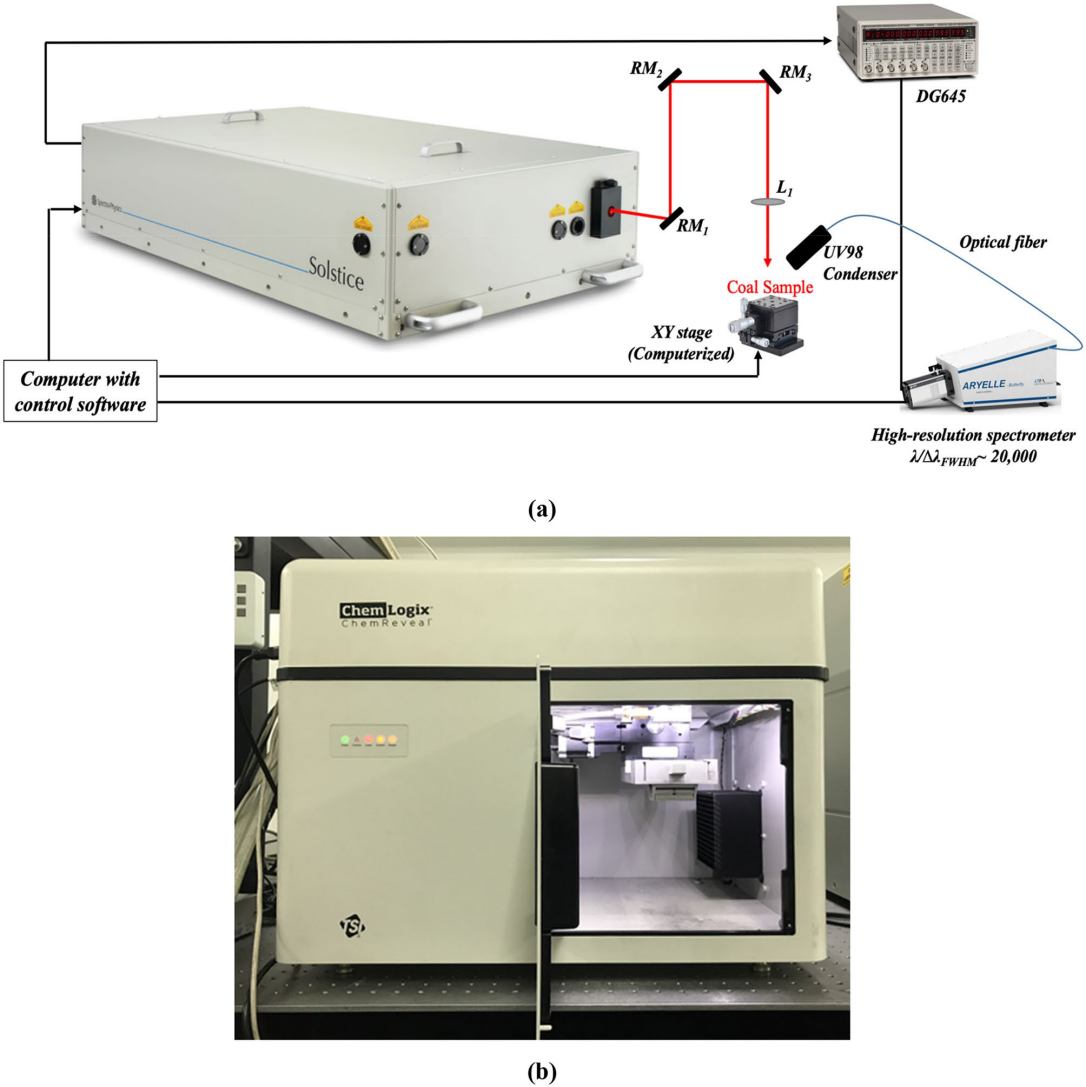
(**a**) Schematic diagram (not to scale) of *fs-Solstice* system under evaluation. RM: ultrafast 730–820 nm reflecting mirrors; L_1_: focusing lens (*f* = 200 mm); (**b**) Image of *ns-Chem* system—an industrially-applied ns-LIBS coal analyzer (offline mode of operation). Photographs courtesy of Sahar Sheta. Copyright 2021.

A one-box ultrafast Ti:Sapphire amplifier (SolsticeAce, Spectra Physics, USA) was targeted for a performance test as a coal analysis system (Fig. 13a). Inside the amplifier box (with 125 × 68 × 29 cm dimensions) are four independent modules: regenerative amplifier, stretcher/compressor, seed laser, and pump laser. Two power supplies and chillers are connected to the system for seed (Mai Tai) and pump (Empower) lasers operation. The system produces laser pulses centered at 800 nm with linear polarization and it is user configurable to operate over: 2–7 mJ pulse energy, 35–120 fs pulse width, and 4–1000 Hz repetition rate. The system employs adjustment-free EternAlign internal optical mounts to maximize long-term stability and operation (Energy stability of < 0.5% rms over 24 h operation). The temperature control unit (TCU) monitors a safe and constant temperature and humidity environment for the Ti:Sapphire crystal. The timing and delay generator (TDG) synchronizes the action of Pockels cells, seed laser, and pump lasers, and provides output signals which allow external instruments to capture laser-firing signals. The output laser beam is a TEM_00_ Gaussian beam profile with beam quality M^2^ of < 1.25 and a diameter of ~ 10 mm. In the studied configuration, TDG was used to externally trigger a laboratory delay generator (DG645, Stanford Research Systems, USA) which enabled precise delays to the laser pulse. The laser beam was first reflected by three 730–820 nm ultrafast mirrors, then focused onto sample surface by a plano-convex lens (Fused silica, f = 200.23 mm, d = 25.4 mm, and 1.6 mm central thickness). The mirrors and focusing lens graphed group delay dispersion of ~ 0 and 36 fs^2^ for wavelengths centered at 800 nm, respectively. An auto-correlator and a power-meter were used to measure the pulse width and energy drifts before and after the experiment. A sample holder was positioned above a computerized XY translation stage (PT1-Z8, Thorlabs, USA). Plasma emission was collected using fused silica collimating lenses (UV98 condenser, LTB, Germany) set at 45^o^ observation angle and connected to an optical fiber. An ICCD echelle-spectrometer (Aryelle-Butterfly, LTB, Germany) with f/10 numerical aperture, 14,000 spectral resolution, and 13 pm wavelength accuracy was employed using its spectral range 192–433 nm. The dark current of the ICCD was subtracted from spectra using Sophi software. For each experimental set, spectrometer was wavelength calibrated using a mercury lamp (DH-3plus, Ocean Tec, USA).

#### Nanosecond coal analyzer (*ns-Chem*)

An offline LIBS coal analyzer (ChemReveal, TSI, USA) was used to evaluate the fs-LIBS system (Fig. 13b). The instrument (with 50 × 60.5 × 50.8 cm dimensions) encloses: laser source, optics train, sample chamber, and spectrometer. The laser source is a Q-switched Nd: YAG laser emitting pulses at 1064 nm wavelength with ~ 8 ns pulse duration and energies vary over 0 to 200 mJ/pulse (± 5% stability) at 1 Hz repetition rate. Robust opto-mechanical design comprises alignment-free reflecting mirrors and focusing lenses and offers adjustable analysis spot size (200–800 µm) with micron-scale precision and control. Sample chamber contains computerized XYZ holder and an optical fiber adjusted 45° with the laser beam. The spectrometer consists of 7 CCD detectors covering the spectral range 190–940 nm with a nominal resolution ~ 0.09 nm and minimum integration time of 1.05 ms.

The aim of this study is to evaluate our fs-LIBS system for coal property analysis application relative to an industrially applied coal analyzer. System evaluation is defined as the comparison of data results to a standard set for the purpose of judging worth of quality^71^ using several objectives to measure effectiveness/performance. In literature, different papers have discussed evaluation of LIBS systems for industrially-related applications. Gaft et al.^10^ evaluated LDS LIBS unit for ash analysis in coal relative to a Coalscan 9500X PGNAA analyzer at Optimum Colliery. Chadwick et al.^72^ evaluated LIBS system for elemental detection of low-ash lignite coal as fuel source. LIBS results were compared relative to standard results set of atomic absorption spectrometry (AAS) at Loy Yang Power. Naes et al.^73^ evaluated LIBS system relative to LA-ICP-MS and μXRF systems for the discrimination of automotive glass. Trevizan et al.^74^ evaluated LIBS system for macronutrients in plant materials relative to ICP-OES after wet acid decomposition. At previous studies, LIBS systems under evaluation were operating at best-achieved results relative to optimized conditions of the standard systems. Accordingly, *ns-Chem* system operates in its factorial-adjusted design customized for coal analysis in power plant. While, the operation parameters of *fs-Solstice* system were selected after an experiment was run using its changeable parameters to achieve best-obtained signal-to-background ratio (SBR), spectral resolution, and to avoid line intensities saturation/interferences. For each fs-pulse energy: 4.9, 5.1, 5.3, 5.7, 5.9, 6.1 and 6.3 mJ, an optimum delay time in the interval of 0–3 µs was chosen. Single, 10, 20, 50, 100, and 200 pulse trains were tested. The selected operation parameters for the fs-LIBS system are listed in Table 9 with the operation parameters of the ns-coal analyzer. All experiments were performed in an atmospheric environment (Lab. temperature was ~ 21–23 °C and humidity was < 20%). The data treatment was performed using Matlab R2018b, Origin, and Excel.

**Table 9:** Operation parameters of *fs-Solstice* and *ns-Chem* systems.

	fs-Solstice*		ns-Chem**	
	Changeable	Fixed	Changeable	Fixed
Laser wavelength, nm	–	800	–	1064
Pulse frequency, Hz	1000	–	–	1
Pulse duration	–	45 fs	–	8 ns
Pulse energy, mJ	5.7	–	90	–
Warming up time, h	–	1.5	–	1
Spot diameter, µm	600	–	200	–
Shots per sample	–	100	–	100
Ablation mode	Fs-train of 100 signals	–	–	Single pulse
Delay time, µs	0.4	–	1	–
Integration time	1 µs	–	–	1.05 ms

*Optimized for best-achieved: SBR, spectral resolution, and no lines saturation/interferences.

**Factorial design based on best-tested: signals’ repeatability and quantitative modeling results.

### Craters morphology

Craters’ surface and depth topologies were studied using focused ion beam scanning electron microscopy FIB-SEM (Lyra 3, TESCAN, Czech Republic) and coherence scanning interferometry CSI (Zygo-NexView, Ametek, USA).
